# Orthogonal Covalent Entrapment of Cargo into Biodegradable Polymeric Micelles via Native Chemical Ligation

**DOI:** 10.1021/acs.biomac.2c00865

**Published:** 2022-08-31

**Authors:** Erik R. Hebels, Felix Bindt, Johanna Walther, Michiel van Geijn, Jimmy Weterings, Qizhi Hu, Claudio Colombo, Rob Liskamp, Cristianne Rijcken, Wim E. Hennink, Tina Vermonden

**Affiliations:** † Division of Pharmaceutics, Utrecht Institute for Pharmaceutical Sciences (UIPS), Utrecht University, 3508 TB Utrecht, The Netherlands; ‡ Cristal Therapeutics, 6229 EV Maastricht, The Netherlands

## Abstract

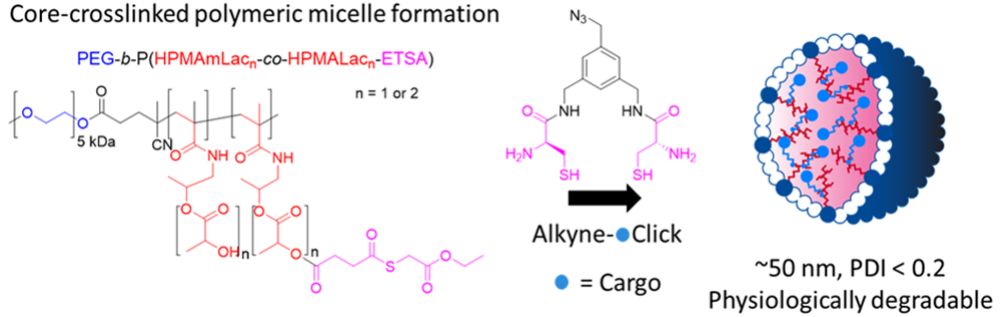

Polymeric micelles (PMs) are promising platforms for enhanced tissue targeting of entrapped therapeutic agents. Strategies to circumvent premature release of entrapped drugs include cross-linking of the micellar core as well as covalent attachment of the drug cargo. The chemistry employed to obtain cross-linked micelles needs to be mild to also allow entrapment of fragile molecules, such as certain peptides, proteins, oligonucleotides, and fluorescent dyes. Native chemical ligation (NCL) is a mild bio-orthogonal reaction between a *N-*terminal cysteine residue and a thioester that proceeds under physiological conditions. Here, we designed a trifunctional cross-linker containing two cysteine residues for the micelle core-cross-linking reaction and an azide residue for ring-strained alkyne conjugation of fluorescent dyes. We applied this approach to thermosensitive methoxypolyethylene glycol-*b*-*N*-(2-hydroxypropyl)methacrylamide-lactate (mPEG-*b*-HPMAmLac*_n_*) based block copolymers of a core-cross-linked polymeric micelle (CCPM) system by attaching thioester residues (using ethyl thioglycolate-succinic anhydride, ETSA) for NCL cross-linking with the trifunctional cross-linker under physiological conditions. By use of mild copper-free click chemistry, we coupled fluorescent dyes, Sulfo.Cy5 and BODIPY, to the core via the azide residue present on the cross-linker by triazole ring formation. In addition, we employed a recently developed cycloheptyne strain promoted click reagent (TMTHSI, CliCr) in comparison to the frequently employed cyclooctyne derivative (DBCO), both achieving successful dye entrapment. The size of the resulting CCPMs could be tuned between 50 and 100 nm by varying the molecular weight of the thermosensitive block and ETSA content. *In vitro* cell experiments showed successful internalization of the dye entrapped CCPMs, which did not affect cell viability up to a polymer concentration of 2 mg/mL in PC3 cells. These fluorescent dye entrapped CCPMs can be applied in diagnostic imaging and the chemistry developed in this study serves as a steppingstone toward covalently entrapped fragile drug compounds with tunable release in CCPMs.

## Introduction

Polymeric micelle (PM) based nanoparticles have emerged in the last decades as a promising platform for tissue targeting of drugs, particularly for applications in cancer therapy. PMs are hallmarked by their colloidal core–shell structure emerging from the constituent amphiphilic block copolymers that spontaneously self-assemble above the critical micellization concentration (CMC). Owing to their synthetic nature, block copolymers used to produce PMs allow for specific versatile tuning of chemical and physical properties, which can be used to tune particle characteristics.^1,2^ Although to present date only a few PM based products have reached the market, several clinical trials making use of new generation PM systems are underway.^3−5^

Tumor directed targeting of nanoparticulate therapeutics, such as PMs, is mostly based on passive accumulation at the tumor site due to leaky vasculature within the tissue and extended circulation time of the nanoparticles compared to the free drug. Such passive accumulation is commonly referred to as the enhanced permeability and retention (EPR) effect^6,7^ and is dependent on a number of factors including the size of the nanoparticle, circulation kinetics as well as tumor and patient type, asserting the need for rational design and patient stratification.^8,9^ Particles need to fall within a size range between ∼5 and 200 nm to avoid clearance by renal excretion and uptake by the mononuclear phagocyte system (MPS).^10,11^ Furthermore, to facilitate deep tumor penetration, particularly for poorly vascularized tumors, a size of around 50 nm or less is preferable.^12−14^ The size of PMs can typically be tuned by varying the molecular weight of the constituent amphiphilic block copolymers.^15,16^ Finally, polyethylene glycol (PEG) surface modification has been employed as a frequently applied stealth polymer block to evade MPS uptake and extend the circulation time of nanoparticle systems.^17−19^

The stability of PM systems in circulation as well as stable entrapment of the drug cargo is crucial for effective drug retention until the target tissue is reached. Due to the inherent equilibrium of amphiphilic block copolymers in the monomeric (or unimers) and assembled PM state, adsorption of these unimers to plasma proteins and excessive dilution drive the disassembly of PMs when administered into circulation.^20^ Disassembly can be circumvented by introducing cross-links between the block copolymers in the assembled polymeric micelle by chemical approaches^21−24^ but also by introduction of additional physical interactions such as stereocomplex formation^25^ or π–π stacking.^26−29^ The same stability consideration applies to the interaction of PMs with the cargo to prevent premature release from the PM system by interaction with competing plasma proteins.^22,30^

Cross-linking of the hydrophobic blocks within the PM to yield core-cross-linked polymeric micelles (CCPMs) is one of the approaches employed to achieve PM stabilization. To this end, a promising CCPM platform (CriPec) was developed and is currently undergoing clinical investigation for the treatment of solid tumors using docetaxel as chemotherapeutic drug cargo.^31−34^ These CCPMs consist of thermosensitive block copolymers that are cross-linked upon micellization by free radical polymerization of methacrylate functionalized side chains present on the core forming part of the block copolymer. Furthermore, the docetaxel cargo, conjugated with a hydrolyzable linker also comprising a methacrylate moiety, is covalently attached to the hydrophobic core using the same free radical chemistry.^35^

Using free radical chemistry, only a select group of molecules including chemotherapeutic agents such as docetaxel as a methacrylate derivative can be conjugated. Many compounds, including new generation pharmaceuticals, are of biological and peptide nature^36^ or have fragile moieties that would be damaged by this approach (particularly lysine, tryptophan and methionine residue containing entities).^37,38^ Furthermore, several fluorescent dyes that are interesting for imaging purposes are also incompatible with free radical chemistry due to their conjugated unsaturated nature.^39^ These challenges highlight the need for compatible cross-linking approaches in PM systems to expand the PM encapsulated drug and dye repertoire.

Native chemical ligation (NCL), introduced by Kent and co-workers in 1994, is a mild bio-orthogonal reaction between a *N*-terminal cysteine residue and a thioester that readily proceeds under physiological conditions.^40,41^ Previously, we have shown the use of NCL for the cross-linking of PMs by complementary reaction of two *N*-isopropylacrylamide based copolymers modified with either cysteine or thioglycolate residues.^42^ Recently, the ultrafast synthesis of block copolymers via NCL was also demonstrated.^43^

Here, we designed a trifunctional cross-linker containing two cysteine residues and an azide functionality to employ NCL as a cross-linking strategy for the formation of CCPMs and simultaneous drug entrapment. The cysteine residues can react with thioglycolate modified copolymers via NCL while the azide functionality can be employed for (copper-free) click chemistry coupling of drugs or fluorescent dye conjugates. Thermosensitive mPEG_5000_-*b-*P(HPMAmLac_1_-*co*-HPMAmLac_2_) block copolymers were functionalized with thioester side chains to allow NCL reaction with the cross-linker under physiological conditions. We investigated the tunability of the size and chemical degradability of the CCPMs as well as the conjugation of dyes Sulfo.Cy5 and BODIPY. Further, we employed the frequently used strain promoted cyclooctyne derivative (BCN) and a recently developed cycloheptyne click reagent (TMTHSI, CliCr)^44^ respectively to demonstrate versatility of the system, and cell experiments were performed *in vitro* to evaluate cell uptake and cytocompatibility.

## Materials and Methods

### Materials

All materials were obtained from Sigma-Aldrich (Zwijndrecht, The Netherlands) unless indicated otherwise. Ethylthioglycolate-succinic acid (ETSA) was synthesized according to a previously published procedure (Figure S1.10 for NMR).^45^ The 2-(methoxy polyethylene glycol)-4,4-azobis(4-cyanopentanoic acid) (mPEG_5000_)_2_-ABCPA) free radical macroinitiator was synthesized according to a previously published procedure.^15^*N*-2-hydroxypropyl methacrylamide monolactate (HPMAmLac_1_) and dilactate (HPMAmLac_2_) as well as 3,3,6,6-tetramethylthiacycloheptyne sulfoximine functionalized 4,4-difluoro-4-bora-3a,4a-diaza-*s*-indacene 650 (BODIPY-TMTHSI) were obtained by custom synthesis from Symeres (Nijmegen, The Netherlands). Dibenzocyclooctyne functionalized sulfonated cyanine 5 (Sulfo.Cy5-DBCO) was obtained from Lumiprobe (Hannover, Germany). All solvents were obtained from Biosolve (Valkenswaard, The Netherlands).

### Synthesis

#### Trifunctional Cross-Linker Synthesis

##### Synthesis Compound **1**

**Scheme 1:**
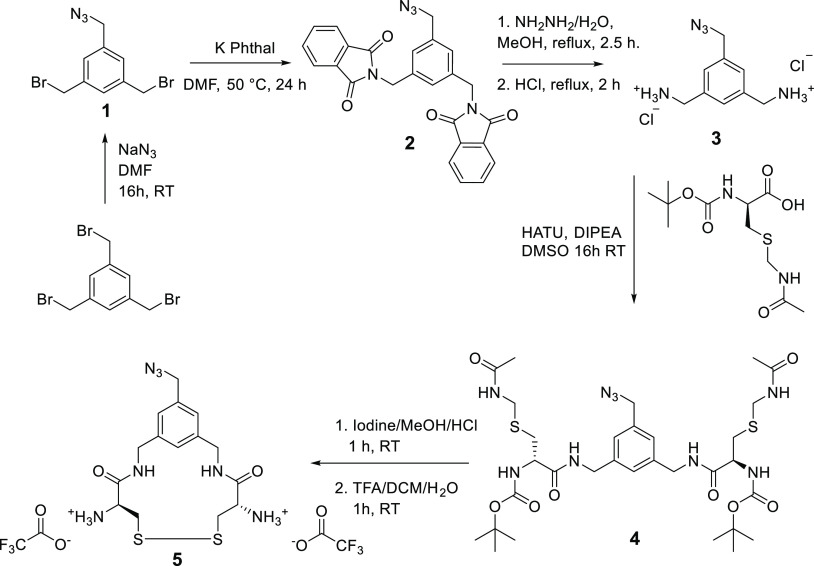
Synthesis of the Azide Containing Di-cysteine Crosslinker (Compound **5**)

In a typical reaction, 1,3,5-tris(bromomethyl)benzene (10.0 g, 28.0 mmol) was dissolved in 20 mL of dimethylformamide (DMF) and the obtained solution was, after addition of sodium azide (2.7 g, 42.0 mmol), stirred for 16 h at room temperature (RT). The reaction mixture was then diluted with 100 mL of ethyl acetate and filtered to remove precipitated salts. The filtrate was then washed (60 mL) twice with 0.1 M HCl, once with brine, dried over anhydrous sodium sulfate, and concentrated using a rotavapor. The residual solvent was removed under vacuum overnight at RT. The obtained oil was then dissolved in diethyl ether to which a few drops of methanol were added and adsorbed onto silica gel on a rotavapor. 1-(Azidomethyl)-3,5-bis(bromomethyl)benzene (compound **1**, Scheme 1) was obtained by two successive silica column purifications using 24:1 hexane:diethyl ether as the eluent (*R*_f_ = ∼0.4 in 9:1 hexane:diethyl ether), yielding 2.6 g (29%) of a faint yellow viscous liquid. ^1^H NMR (400 MHz, chloroform-*d*): δ 7.39 (s, 1H), 7.28 (s, 2H), 4.47 (s, 4H), 4.37 (s, 2H).^46^

##### Synthesis Compound **2**

Intermediate compound **2** (azidomethyl-3,5-di(phthalamidomethyl)benzene) was obtained by dissolving compound **1** (2.1 g, 6.6 mmol) in 20 mL of dry DMF and stirring with potassium phthalimide (2.8 g, 15.1 mmol) for 16 h at 50 °C under an argon atmosphere. The mixture was diluted in 150 mL of dichloromethane (DCM) and filtered to remove precipitated KBr. The filtrate was washed (100 mL) twice with Milli-Q water, once with brine, dried over anhydrous sodium sulfate, concentrated on a rotavapor and residual solvent was removed under vacuum at RT, yielding 2.8 g (94%) of a white solid. ^1^H NMR (400 MHz, chloroform-*d*): δ 7.86–7.80 (m, 4H), 7.73–7.67 (m, 4H), 7.46 (s, 1H), 7.24 (s, 2H), 4.81 (s, 4H), 4.27 (s, 2H).

##### Phthalimide Deprotection of Compound **2** to **3**

Compound **2** (2.8 g, 6.2 mmol) was subsequently deprotected to yield compound **3** (azidomethyl-3,5-di(aminomethyl)benzene) using hydrazine hydrate (0.9 mL, 13.6 mmol) in 100 mL of methanol and boiling under reflux for 3 h. After cooling to RT, 150 mL of Milli-Q water was added followed by 25 mL of concentrated HCl, and the mixture was boiled under reflux for 2 h. The white suspension was then filtered to remove salts, concentrated using a rotavapor, and dried overnight under vacuum yielding a white solid HCl salt containing inorganic salt impurities, 2.3 g. ^1^H NMR (400 MHz, DMSO-*d*_6_): δ 8.66 (s, 6H), 7.61 (s, 1H), 7.51 (s, 2H), 4.48 (s, 2H), 3.99 (q, *J* = 5.8 Hz, 4H).

##### Synthesis Compound **4**

Boc-Cys(Acm)-OH residues were coupled to the crude compound **3** (0.60 g) to obtain the protected form of the dicysteine cross-linker, compound **4**. Boc-Cys(Acm)-OH (1.5 g, 5.0 mmol) was dissolved in 5 mL of dry DMSO, after which hexafluorophosphate azabenzotriazole tetramethyl uronium (HATU) (1.9 g, 5.0 mmol) and *N*,*N*-diisopropylethylamine (DIPEA) (7.9 mL, 45.4 mmol) were added, and the mixture was stirred for 15 min. Compound **3** was added and the reaction mixture was stirred for 16 h at RT. The mixture was diluted with 100 mL of chloroform, washed (100 mL) twice with 0.1 M HCl, twice with saturated NaHCO_3_, once with brine, dried over anhydrous sodium sulfate, concentrated using a rotavapor, and dried under vacuum overnight. The obtained solid was dissolved in methanol, dispersed and subsequently concentrated in silica gel and purified by silica column chromatography employing 19:1 DCM:MeOH as the eluent (*R*_f_ = ∼0.3), yielding 0.79 g (70% yield relative to compound **2**). ^1^H NMR (400 MHz, DMSO-*d*_6_): δ 8.46 (t, *J* = 6.2 Hz, 2H), 8.36 (t, *J* = 6.0 Hz, 2H), 7.16–7.09 (m, 3H), 6.96 (d, *J* = 8.4 Hz, 2H), 4.38 (s, 2H), 4.31–4.11 (m, 10H), 2.94–2.61 (m, 4H), 1.80 (s, 6H), 1.37 (s, 18H).

##### Acetamidomethyl (Acm) Deprotection of Compound **4**

Compound **4** was deprotected to obtain the disulfide cross-linker compound **5**. For this, compound **4** (0.38 g, 0.51 mmol) was dissolved in 9 mL of methanol. To this solution, 18 mL of 0.1 M HCl and subsequently 9 mL of 0.4 M I_2_ in methanol were added to deprotect the thiol Acm protecting groups. The reaction mixture was stirred for 1 h at RT, after which excess iodine was removed by addition of a 1 M aqueous ascorbic acid solution until the solution turned to a white suspension. Chloroform was added to the mixture and concentrated on a rotavapor at 40 °C. The formed white product precipitate was vacuum filtered. Additional chloroform and water were added to wash the precipitate and this filter process was repeated 5 times. ^1^H NMR (400 MHz, DMSO-*d*_6_): δ 8.46 (s, 2H), 7.15–7.00 (m, 5H), 4.34 (s, 2H), 4.30–4.16 (m, 6H), 3.13–2.79 (m, 4H), 1.36 (s, 18H).

##### Tert-butoxycarbonyl (Boc) Deprotection of Compound **4**

To remove the Boc protecting group, the precipitate was dissolved in 20 mL of DCM/TFA/water 49:49:2 and stirred at RT for 1 h. The mixture was concentrated using a rotavapor, followed by coevaporation first with DCM and then hexane, after which residual solvents were removed under vacuum overnight, yielding 0.29 g (91% relative to compound **4**) of a yellowish crystalline solid. ^1^H NMR (400 MHz, DMSO-*d*_6_) δ 9.22 (s, 2H), 8.48 (s, 6H), 7.20 (s, 1H), 7.17 (s, 2H), 4.41 (s, 2H), 4.30–3.92 (m, 6H), 3.40–2.98 (m, 4H). HR-MS: Expected mass for C_15_H_22_N_7_O_2_S_2_^+^ 396.1271; found, 396.1271.

#### Thermosensitive Block Copolymer Library Synthesis and Thioester Modification

**Scheme 2:**
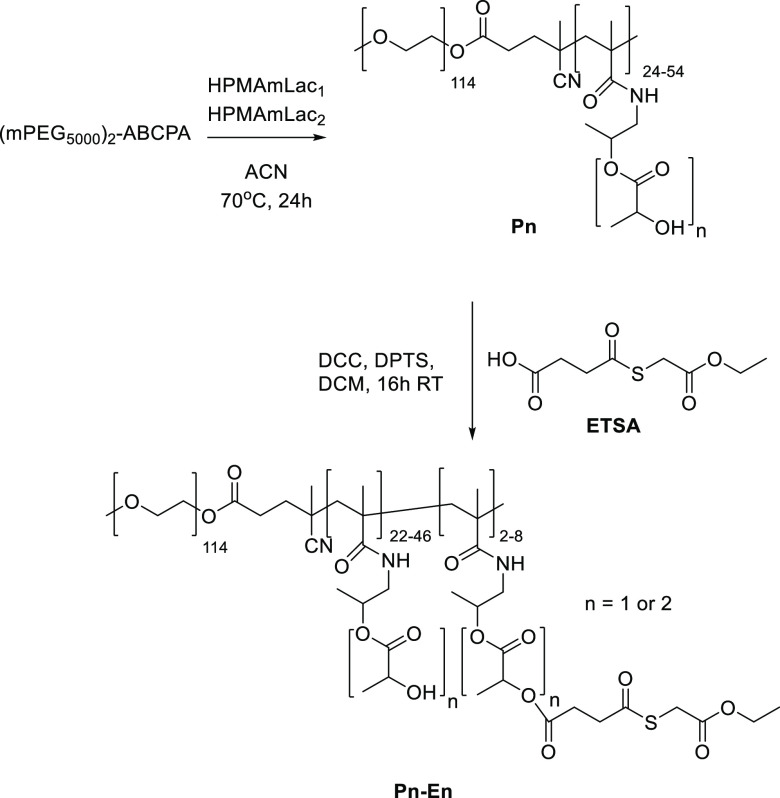
Synthesis of mPEG_5000_-*b*-P(HPMAmLac_1_-*co*-HPMAmLac_2_) Polymers (polymer **Pn**) by Free Radical Polymerization Using a (Methoxy polyethylene glycol)_2_-4,4-azobis(4-cyanopentanoic acid) ((mPEG_5000_)_2_-ABCPA) Macroinitiator and Subsequent ETSA Coupling to Attain Polymer **Pn**-**En**

A five membered library of mPEG_5000_-*b*-P(HPMAmLac_1_-*co*-HPMAmLac_2_) block copolymers (polymer **P** (monomer to initiator ratio), Scheme 2) was synthesized by free radical polymerization following a previously published procedure.^16^ Briefly, varying amounts (160–480 mg) of the (mPEG_5000_)_2_-ABCPA macroinitiator were weighed into a Schlenk tube followed by HPMAm-monolactate (HPMAmLac_1_, 274 mg) and HPMAm-dilactate (HPMAmLac_2_, 326 mg). ACN was added to result in a 300 mg/mL monomer concentration. These amounts resulted in a fixed ratio of 53:47 HPMAmLac_1_:HPMAmLac_2_, and feed molar ratios of monomer/initiator of 150, 125, 100, 75, and 50 were used. The tubes were sealed by a rubber septum and 5 freeze–vacuum–thaw cycles were applied, backflushed with nitrogen and placed into a preheated oil bath at 70 °C for 24 h. The reaction mixtures were cooled to RT, and the obtained products were precipitated 3 times in diethyl ether and placed under vacuum overnight, yielding white solids. The synthesized polymers were characterized by gel permeation chromatography (GPC) and NMR (Figure S1.9).

The different block copolymers were functionalized in a subsequent reaction to obtain mPEG_5000_-*b*-P(HPMAmLac_*n*_-*co*-HPMAmLac_*n*_-ETSA) block copolymers (polymer **Pn-E**(ETSA functionalization percentage)). Stock solutions of thioester ETSA (50 mg/mL), DPTS (20 mg/mL), and DCC (50 mg/mL) in dry DCM were prepared. Polymer **Pn** was weighed and dissolved in dry DCM (final concentration of 100 mg/mL), followed by addition of ETSA with a feed ratio of 10 and 15 mol % relative to HPMAmLac_*n*_ functionalization, 0.1 equiv (to ETSA) of DPTS and finally 1.1 equiv (to ETSA) of DCC. The reaction mixtures were stirred at RT for 24 h. The mixtures were then filtered using a 0.2 μm PTFE syringe filter to remove precipitated DCU. Subsequently, the polymers were precipitated twice in diethyl ether and dried under vacuum overnight. The synthesized polymers were characterized using GPC and NMR analysis (Figure S1.11).

#### Core-Cross-Linked Polymeric Micelle (CCPM) Formation and Purification

The mPEG_5000_-*b*-P(HPMAmLac_*n*_-*co*-HPMAmLac*_n_*-ETSA) polymers (polymer **Pn-En**) were dissolved in a phosphate buffer (100 mM Na_2_HPO_4_, adjusted to pH 7.4 using HCl) to 20 mg/mL while stirring in an ice bath. The cross-linker (compound **5**) was dissolved in DMSO (75 mg/mL), and 3 equiv (to cross-linker) of tris(2-carboxyethyl)phosphine (TCEP) in DMSO (112.5 mg/mL) was added to reduce the disulfide bonds. After 20 min, the cross-linker/TCEP mixture (having 1.5 equiv of cysteine residues relative to the ETSA residues on the polymer chains) was placed in a water bath preheated to 37 °C while stirring and the polymer solution (typically 1 mL) was then added. After 1 h of cross-linking, the obtained core-cross-linked micelles were filtered using 0.2 μm RC syringe filters (rinsed with additional phosphate buffer resulting in a 2-fold dilution) and subsequently purified using a G-25 sephadex packed 5 mL HiTrap desalting column with an *M*_w_ cutoff of 5000 Da (GE healthcare, Uppsala, Sweden).

##### Bicyclo[6.1.0]non-4-yn-9-ylmethanol (BCN-OH) Copper-Free Click Conjugation with Cross-Linker (Compound **6**)

**Scheme 3:**
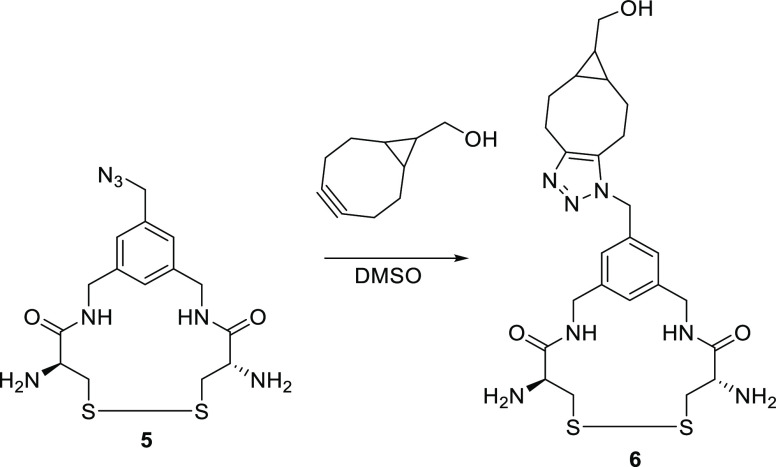
One Pot Reaction of the Trifunctional Crosslinker with BCN-OH BCN-OH is clicked to the trifunctional cross-linker (compound **5**) in DMSO and dried for analysis to confirm the azide based coupling.

Cross-linker compound **5** (11 mg, 17 μmol) was weighed into a glass vial followed by BCN-OH (3.5 mg, 23 μmol) and 100 μL of DMSO (see Scheme 3). The reaction mixture was left stirring for 2 h RT, after which the product in the vial was lyophilized overnight. The dried solid was analyzed by ^1^H NMR, infrared (IR) spectroscopy and electrospray ionization mass spectrometry (ESI-MS) (Figures S6.1 and S2.2).

##### Sulfo.Cy5-DBCO Loaded CCPM Formation (Compound **7** Construct)

**Scheme 4:**
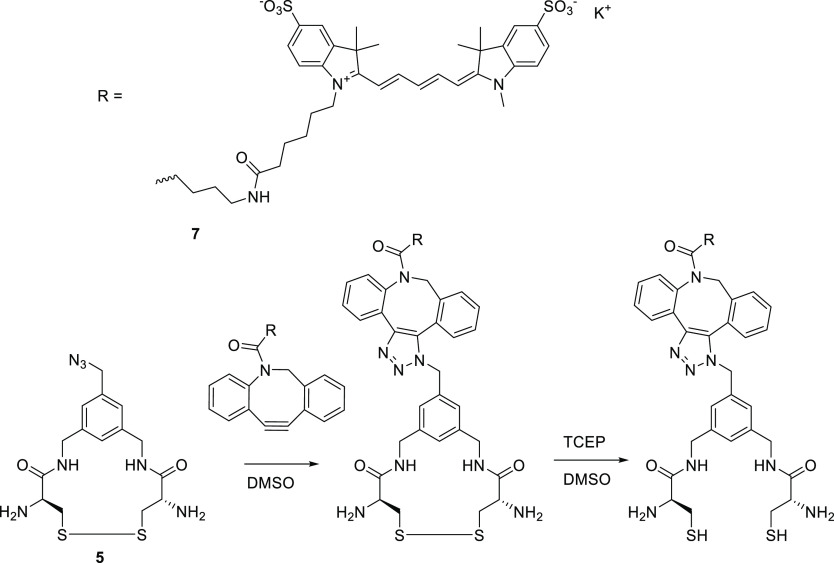
One Pot Reaction of the Trifunctional Cross-Linker with the DBCO Functionalized Sulfo.Cy5 Sulfo.Cy5-DBCO is clicked to the trifunctional cross-linker (compound **5**), after which the disulfide bonds are reduced using TCEP for NCL cross-linking of the cross-linker-Cy5 conjugate with **Pn-En** to obtain CCPMs.

Prior to CCPM formation, a Sulfo.Cy5-cross-linker conjugate was obtained in a one pot reaction as shown in Scheme 4. The cross-linker (compound **5**) was dissolved in DMSO (75 mg/mL). Sulfo.Cy5-DBCO was dissolved in DMSO to 10 mg/mL, 2% mass relative to the polymer added to the cross-linker (6.5 mol % relative to cross-linker) and the mixture was stirred for 1 h at RT. TCEP (dissolved to 112.5 mg/mL in DMSO) was then added in 3-fold molar excess to the mixture to reduce the disulfide bonds. After 20 min, the mixture was placed in a water bath preheated to 37 °C. mPEG_5000_-*b*-P(HPMAmLac*_n_*-*co*-HPMAmLac*_n_*-ETSA) (polymer **Pn-En**), dissolved in a phosphate buffer (100 mM Na_2_HPO_4_, adjusted to pH 7.4 using HCl) to 20 mg/mL on an ice bath, was added to the cross-linker/Sulfo.Cy5/TCEP mixture and the reaction mixture was stirred for 1 h. The obtained CCPMs were purified as described above.

##### BODIPY-TMTHSI Loaded CCPM Formation (Compound **8** Construct)

**Scheme 5:**
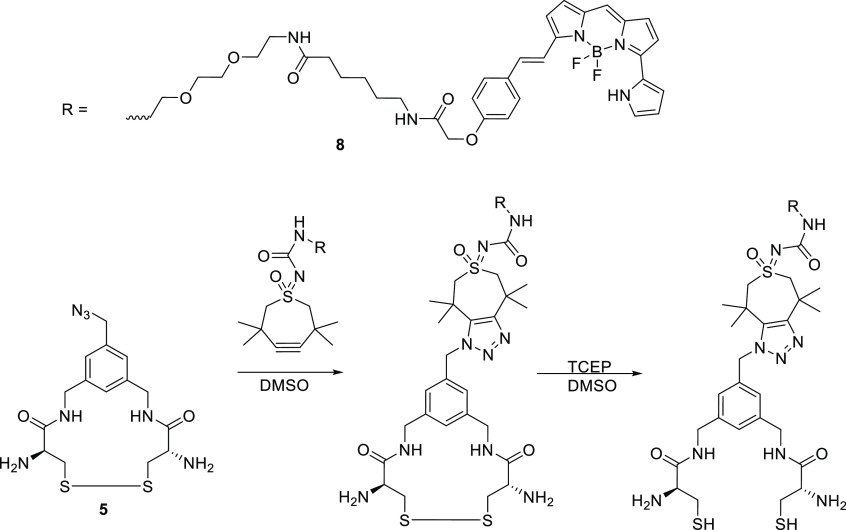
One Pot Reaction of the Trifunctional Cross-Linker with the TMTHSI Functionalized BODIPY Dye BODIPY-TMTHSI is clicked to the trifunctional cross-linker (compound **5**), after which the disulfide bonds are reduced using TCEP for NCL cross-linking of the cross-linker-BODIPY conjugate with **Pn-En** to obtain CCPMs.

Prior to CCPM formation, a BODIPY-cross-linker conjugate was obtained in a one pot reaction as shown in Scheme 5. The same procedure as described above for the Sulfo.Cy5-cross-linker conjugate was employed.

### Cross-Linker, Polymer, and Particle Characterization

#### Nuclear Magnetic Resonance (NMR) Spectroscopy

^1^H, ^13^C, and ^1^H–^13^C heteronuclear single quantum correlation (HSQC) NMR spectra were recorded on an Agilent 400-MR NMR spectrometer (Agilent Technologies, Santa Clara, USA). Residual solvent peaks of CDCl_3_ (δ = 7.26 ppm) or DMSO-*d*_6_ (δ = 2.50 ppm) were used to calibrate chemical shifts.

#### IR Spectroscopy

IR spectra were recorded with solid samples using a ATRU equipped Spectrum 2 (PerkinElmer, Llantrisant, UK) and reported in cm^–1^.

#### Mass spectrometry (MS)

Three methods of MS were employed in this work. High resolution (HR)-MS was performed on a 6560 Ion Mobility Q-TOF LC/MS (Agilent technologies, Santa Clara, USA). Normal resolution electrospray ionization (ESI)-MS measurements were performed on a microTOF-Q II (Bruker). Matrix assisted laser desorption ionization (MALDI)-MS analyses were performed on a Ultraflextreme (Bruker), employing α-cyano-4-hydroxycinnamic acid as the matrix.

#### Thin Layer Chromatography (TLC)

TLC was carried out using aluminum bound silica plates obtained from Merck Darmstadt (SiO_2_, Kieselgel 60 F254). Compounds were visualized by UV detection at 254 nm and, where applicable, stained with ninhydrin in DCM to visualize amines/amides or 10% triphenylphosphine in DCM followed by ninhydrin to visualize azides.

#### Gel Permeation Chromatography (GPC)

GPC for polymer analysis was performed using an Alliance 2695 (Waters) chromatography system with two PLgel 5 μm mixed-D columns (Polymer Laboratories) in series at a column temperature of 65 °C and employing a refractive index detector. DMF supplemented with 10 mM LiCl was employed as the eluent with an elution rate of 1 mL/min. Sample concentration was typically 5 mg/mL, and PEGs of narrow and defined molecular weights obtained from PSS (Germany) were used as calibration standards. Recording of data and calculations of molecular weights were done with Waters Empower 32 software.

For the GPC analysis of empty CCPMs, serially connected TSKGel G5000PWXL 10 μm (first column) and G6000PXWL 13 μm (second column) 7.8 × 300 mm columns (Tosoh Bioscience, Griesheim, Germany) were employed at a column temperature of 25 °C and isocratic flow of 0.3 mL/min of 100 mM NaNO_3_ supplemented with 10 mM Na_2_HPO_4_/NaH_2_PO_4_ (pH 7.2). A Waters 2487 UV detector was employed measuring absorbance at 235 nm.

#### High Performance Liquid Chromatography (HPLC)

HPLC analysis was performed using an Alliance 2695 chromatography system with an XBridge C18 column (5 μm, 4.6 × 150 mm, Waters) at a column temperature of 30 °C and employing an Alliance 2487 ultraviolet (UV) detector at 210 and 650 or 700 nm. Acetonitrile/water supplemented with either 0.1% perchloric acid or 0.1% formic acid was used as the eluent at a flow of 1 mL/min and a gradient of 5 to 95% acetonitrile over 20 min.

#### Ultrahigh Performance Liquid Chromatography (UHPLC)

UHPLC analysis for lactic and succinic acid quantification was performed using an Acquity (Waters) chromatography system with an HSS T3 column (1.8 μm, 2.1 × 150 mm, Waters) at a column temperature of 30 °C and employing a Waters TUV detector at 210 nm. A KH_2_PO_4_ buffer (10 mM, pH = 2.5) was used as the isocratic eluent at a flow of 0.85 mL/min for 2.5 min followed by an increasing gradient of acetonitrile supplemented with 0.1% phosphoric acid from 0% to 90% over 1 min.

#### Total Polymer Content Analysis

Total polymer content of empty micelles before and after purification was determined through lactic acid concentration by UHPLC after hydrolysis as reported previously.^35^ Briefly, 100 μL of the diluted micellar dispersion was incubated with 100 μL of NaOH (6 M) for 2 h at 60 °C followed by the addition of 200 μL of HCl (6 M). After addition of 200 μL of the eluent (10 mM KH_2_PO_4_, pH = 2.5) for further dilution, samples were run on UHPLC as described above. Sodium lactate was employed as a reference standard to determine the lactic acid concentration.

The total polymer content was calculated as follows: Amount of polymer = measured amount of lactic acid × (*M* + 5000)/ [90.08 × (*m* + 2*n*)], where M is the *M*_n_ of the thermosensitive block P(HPMAmLac_*n*_); *m* and *n* are the number of repeat units of HPMAmLac_1_ and HPMAmLac_2_ in the block copolymer respectively (determined by ^1^H NMR). The molecular weight of lactic acid is 90.08 g/mol.

#### Cloud Point (CP) Measurement

The CPs of the different thermosensitive polymers in a phosphate buffer (100 mM Na_2_HPO_4_, adjusted to pH 7.4 using HCl, 5 mg/mL polymer) were determined by measurement of light scattering at a 90° angle upon the onset of opalescence. Scattered light intensity was measured using a Jasco FP-8300 spectrophotometer employing an excitation and emission wavelength of 550 nm with 1 nm slit width and a response time of 1 s. Temperature was ramped from 2 to 50 °C at 1 °C per minute.

#### Dynamic Light Scattering (DLS)

The size of the CCPMs was determined by DLS using a Malvern Zetasizer nano series ZS90 with a measurement angle of 90°. Measurements were typically carried out at 2, 25, and 37 °C. Unless stated otherwise, the concentration of the micellar dispersions was approximately 7.5 mg/mL in phosphate buffer (100 mM Na_2_HPO_4_, adjusted to pH 7.4 using HCl). For the S.Cy5 and BODIPY loaded CCPMs, a narrow band filter was employed with a 100× sample dilution in Milli-Q to mitigate interference from the dyes.

#### CCPM Physical Stability

The physical stability of the CCPMs was investigated in the presence of increasing surfactant sodium dodecyl sulfate (SDS) concentration. CCPMs were prepared as described above using the P100-E15 polymer and filtered with a 0.2 μm syringe filter. Non-cross-linked control micelles were prepared using the same procedure but without the addition of the cross-linker. The samples (400 μL) were spiked stepwise with either 10 or 20 μL of SDS (20% solution), gently mixed, and measured using DLS at 25 °C.

#### CCPM Hydrolytic Stability

Size exclusion purified empty CCPMs were 5× diluted in a phosphate buffer (100 mM Na_2_HPO_4_, adjusted to pH 7.4 using HCl) to a concentration of 1.5 mg/mL, incubated at 37 °C, and degradation was monitored by the derived count rate (DCR), Z-Ave, and PDI measured by DLS on a Zetasizer Nano S (Malvern Panalytical). Measurements were terminated when the DCR dropped below 5% initial kcps or the PDI exceeded 0.5. Lactic acid release was quantified using the UHPLC method described above, injecting 20 μL of a 7.5 mg/mL micellar suspension diluted with 10 μL of 1 M HCl and 20 μL of Milli-Q water. Total lactic acid content was determined by hydrolyzing 20 μL of a micellar suspension overnight with 10 μL of 1 M NaOH, followed by addition of 20 μL of 1 M HCl.

### Cytotoxicity and Internalization Studies

#### Cell Culture

Prostate cancer cells obtained from American Type Culture Collection (PC3 ATCC CRL-1435) were cultured and maintained at 37 °C with McCoy’s medium supplemented with 10% fetal bovine serum (FBS) in an incubator regulated with 5% CO_2_, 95% air and saturated humidity. Cells were passaged every 2–4 days upon reaching 80% confluency using trypsin ethylenediaminetetraacetic acid (trypsin-EDTA).

#### Cell Viability

PC3 cells were plated into black polystyrene 96 well plates (with microclear bottom, Greiner #655090) at a density of 1.5 × 10^4^ cells per well and incubated for 24 h at 37 °C. The medium was aspirated, and 100 μL dilutions of purified BODIPY loaded CCPMs in McCoy’s medium supplemented with 10% FBS were added in triplicate. After 24 h, 20 μL of MTS staining solution (CellTiter 96 AQ_ueous_, Promega) was added. Following a 1 h incubation, absorbance intensity (490 nm) was recorded using a Mithras plate reader. Data were background subtracted and normalized using medium only wells and untreated cells respectively of the same plate.

#### Cellular Uptake

##### Microscopy

PC3 cells were plated into a black polystyrene 96 well plate (Greiner #655090) at 60% confluency per well in McCoy’s medium with 10% FBS and supplemented with 1% penicillin/streptomycin. The following day, nonlabeled and BODIPY loaded CCPMs were added to the cells in triplicate at a final concentration of 0.1 mg/mL and incubated for 24 h. Prior to confocal microscopy, the cells were treated with 2 μg/mL Hoechst 33342 for 10 min in an incubator at 37 °C, 5% CO_2_. The cells were imaged in OptiMEM on Yokogawa CV 7000 Microscope (60× water immersion objective lens).

##### Fluorescence-Activated Cell Sorting (FACS)

PC3 cells were plated into 12 well plates with a cell confluency of 60% per well and incubated for 24 h at 37 °C, 5% CO_2_ in an incubator. Nonlabeled and BODIPY loaded CCPMs were diluted to 0.1 mg/mL with McCoy’s medium supplemented with 10% FBS and 1% penicillin/streptomycin and after aspiration of culture medium 1000 μL of CCPM dispersion was added to the cells in triplicate. After a 24 h incubation, the treatment medium was removed and the cells washed with PBS. The cells were then harvested using trypsin, transferred into 5 mL polystyrene tubes, and resuspended in PBS supplemented with 5% FBS. Cell associated fluorescence was detected using a FACSCanto II flow cytometer (BD canto II) with 1 × 10^4^ cells per sample.

## Results and Discussion

### Di-cysteine, Azide Functionalized Cross-Linker Synthesis

We designed a trifunctional cross-linker to employ NCL as a cross-linking strategy for the formation of core-cross-linked polymeric micelles (CCPMs) and simultaneous encapsulation of model compounds (fluorescent dyes) under mild conditions. The cross-linker contains two cysteine residues needed for NCL, connected via a benzylic core that also contains an azide moiety for use as a click chemistry handle (compound **5**, Scheme 1). First, a nucleophilic substitution with sodium azide was employed to introduce an azide moiety onto 1,3,5-tris(bromomethyl)benzene. A statistical mixture of mono, di and tri azide-substituted compounds was obtained and compound **1** was isolated after column purification. The close retention times of the statistically substituted products required strenuous fractionation, where particularly unreacted 1,3,5-tris(bromomethyl)benzene was the most difficult impurity to separate from compound **1**. Therefore, instead of 1.0 equiv to target a single substitution, 1.5 equiv of sodium azide were employed to reduce the amount of starting reagent remaining. The identity of compound **1** was confirmed by ^1^H NMR analysis (Figure S1.1).

In the next step, Gabriel synthesis^47^ was employed to substitute the two remaining benzyl bromines with amine functionalities (compound **3**), which involved an intermediate compound **2**: (azidomethyl-3,5-di(phthalamidomethyl)benzene). The phthalimides were introduced by a nucleophilic substitution of the bromides with potassium phthalimide in DMF and after purification, the identity of compound **2** was confirmed by NMR (Figure S1.2). The phthalimide moieties were subsequently converted to amines by reflux boiling with hydrazine hydrate followed by HCl treatment to attain compound **3**. Since multiple attempts to extract compound **3** from the aqueous solution failed, this compound was isolated as a crude HCl salt. The identity of compound **3** was confirmed by ^1^H NMR (Figures S1.3 and S1.4)

We coupled protected cysteine residues to the amines of compound **3** (azidomethyl-3,5-di(aminomethyl)benzene) using the coupling reagent HATU. A great excess of base (20 equiv DIPEA) had to be added to neutralize remaining HCl salts in compound **3**. Compound **4** was obtained via silica column purification and its structure was confirmed by ^1^H NMR analysis (Figure S1.5). A good yield of 70% of compound **4** for two steps starting from compound **2** was obtained.

The final cross-linker (compound **5**) was obtained by deprotecting compound **4**. We removed the Acm protecting group first by use of iodine oxidation. Successful Acm deprotection of compound **4** was confirmed by ^1^H NMR analysis (Figure S1.6). Subsequently, Boc deprotection was carried out using a DCM/TFA/water mixture. After this deprotection step, the reaction mixture was concentrated and finally placed under vacuum yielding compound **5** as TFA salt in a quantitative yield. The identity of compound **5** was confirmed by ^1^H NMR, HR-MS, IR, and HSQC NMR analysis (Figures S1.7, S1.8, S2.1, and S6.1).

### Polymer Synthesis

We synthesized thermosensitive mPEG_5000_-*b-*P(HPMAmLac_1_-*co*-HPMAmLac_2_) block copolymers (polymer **Pn**) with varying molecular weights of the thermosensitive P(HPMAmLac*_n_*) block via free radical polymerization as described in detail previously.^16^ By varying the molar ratio of monomer/macroinitiator (150, 125, 100, 75, and 50 for **P150**, **P125**, **P100**, **P75**, and **P50,** respectively), polymers with varying P(HPMAmLac_*n*_) block lengths and constant mPEG_5000_ length were obtained (Table 1). Generally, a conversion of around 80% was achieved after 24 h of polymerization. Owing to the nature of free radical polymerization, dispersity values between 1.4 and 1.7 were found, as also reported previously.^16^

**Table 1:** Characteristics of mPEG_5000_-*b*-P(HPMAmLac_1_-*co*-HPMAmLac_2_) Block Copolymers before and after Functionalization with ETSA

	eq HPMAmLac_**1**_ per PEG Chain		eq HPMAmLac_**2**_ per PEG Chain						ETSA 10mol %Feed		ETSA 15mol %Feed	
	Feed	Obtained^a^	Feed	Obtained^a^	Lac_1_/Lac_2_ Ratio	*M*_n_ (kDa)^a^	PDI^b^	Cloud Point (°C)	Obtained(mol %)^a^	Cloud Point (°C)	Obtained(mol %)^a^	Cloud Point (°C)
P150	40	31	35	23	1.37	18.2	1.48	35	7.5	10	13.9	3
P125	33	28	29	22	1.27	17.4	1.69	35	8.1	10	14.0	3
P100	27	22	24	19	1.15	15.2	1.70	31	6.5	13	14.1	5
P75	20	16	18	13	1.21	12.1	1.66	31	7.1	13	15.4	5
P50	13	13	12	11	1.18	11.1	1.43	34	7.4	14	16.0	8

a Determined by NMR.

b Determined by GPC.

The different polymers were functionalized with both 10 and 15 mol % ETSA (relative to the amount of HPMAmLac_*n*_ groups) to provide thioester handles for the native chemical ligation reaction, yielding polymers **Pn-E10 and Pn-E15**, respectively (Table 1). By using 1.1 equiv of the DCC coupling reagent, efficient coupling of ETSA to the hydroxyl groups of the P(HPMAmLac*_n_*) block could be achieved as was identified by NMR (Figure S1.11). Additional confirmation of ETSA content was determined by UHPLC quantification of succinic acid after basic hydrolysis of **P100-E15**, finding back 12 mol % ETSA content (Figure S4.1 for chromatogram). Furthermore, free ETSA (that could interfere with the polymer chain cross-linking) was not detected in the purified mPEG_5000_-*b-*P(HPMAmLac_*n*_-*co*-HPMAmLac*_n_*-ETSA) using HPLC analysis (Figure S3.1).

The CP of the unmodified polymers is in agreement with previous findings^16^ and modification with ETSA resulted in a decrease in CP (from around 31–35 °C to 3–14 °C) due to increased hydrophobicity of the thermosensitive polymer block.

### Core-Cross-Linked Micelle Formation

CCPMs were prepared using the 6.5–8.1 and 13.9–16.0 mol % (relative to HPMAmLac_*n*_) ETSA modified polymers (**Pn-En**, Table 1) in combination with cross-linker **5**. The cross-linker was treated with an excess of TCEP to break the disulfide bonds and introduce free thiols for the NCL reaction. During micellization at 37 °C for 1 h, reaction of the cysteine groups of the cross-linker with the thioester functional groups present on the mPEG_5000_-*b*-P(HPMAmLac_*n*_-*co*-HPMAmLac_*n*_-ETSA) polymers resulted in the formation of amide bonds. Thioglycolate was released as side product and subsequently removed by size exclusion chromatography, as confirmed by HPLC analysis (Figure S3.2).

Decreasing the molecular weight of the polymer chains decreased the size of the obtained CCPMs (Table 2), following a similar trend observed previously for the methacrylated derivative of this PEG-P(HPMAmLac_*n*_) polymer system.^16^ Furthermore, increasing the ETSA content from 6.5–8.1 to 13.9–16.0 mol % resulted in substantially reduced micelle size, likely due to an increased hydrophobicity and consequently lower CP of the P(HPMAmLac_*n*_) block causing a denser hydrophobic core.

**Table 2:** Characteristics of CCPMs Formed from the mPEG_5000_-*b*-P(HPMAmLac_*n*_-*co*-HPMAmLac*_n_*-ETSA) Polymers Libraries^a^

	Z-Ave (nm)	PDI	Derived Count Rate (kcps)
P150-E10	119	0.11	103692
P150-E15	79	0.12	56642
P125-E10	115	0.15	93010
P125-E15	73	0.09	62030
P100-E10	68	0.07	38570
P100-E15	50	0.05	20466
P75-E10	61	0.08	16351
P75-E15	45	0.04	12168
P50-E10	57	0.18	6571
P50-E15	42	0.06	7110

a DLS measurements were done at 25 °C.

Particle size plays a crucial role in nanocarrier systems intended for tumoral targeting and exploiting prolonged circulation kinetics and tumor disposition via the EPR effect.^6−14^ Nanoparticles with a size of around 50 nm showed effective tumor accumulation and penetration.^12−14^ Since the decrease in the hydrodynamic diameter (Z-Ave) of the CCPMs levels off (40–50 nm) for polymers smaller than P100-E15 (15.2 kDa), the P100-E15 particle formulation was selected as the lead formulation for further experiments. This choice was made to maximize the number of ETSA moieties available for cross-linking while still obtaining small CCPM sizes. The subsequent resyntheses of the P100-E15 based CCPMs (including resynthesized polymers) resulted in consistent Z-Ave values of between 47 and 53 nm, with a nearly neutral ζ-potential of −4 mV, as expected for PEGylated particles.

The total polymer content of the P100-E15 CCPM dispersion before and after purification by filtration and size exclusion procedure was determined. The purified CCPM sample had a polymer concentration of 7.5 mg/mL, which is very close to the expected value of 7.0 mg/mL based on dilution factors, indicating no significant losses of polymer during purification (Figure S4.2). Furthermore, the single peak found from SEC of CCPMs demonstrates that no free polymer chains remained in the micellar dispersion and thus confirming complete polymer incorporation into the micelles (Figure S5.1). The depletion of the cross-linker peak in the crude sample shown by HPLC (Figure S3.2) also demonstrates complete incorporation into the system.

**Figure 1:**
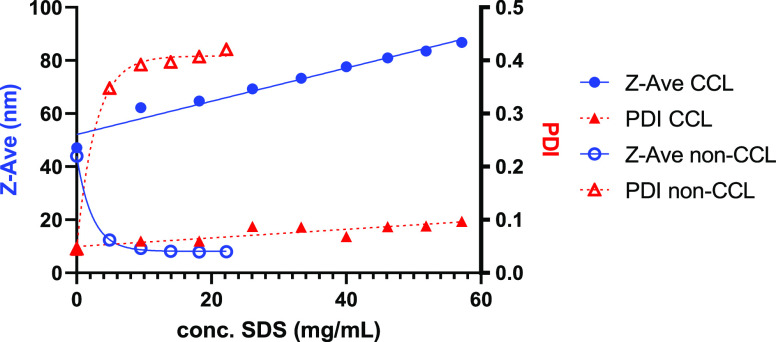
Effect of increasing concentrations of SDS on cross-linked micelles (filled shapes) and non-cross-linked micelles (hollow shapes) as measured by DLS at 25 °C.

The stability of the CCPMs was investigated using DLS experiments by incubating the micellar dispersions with increasing concentrations of SDS, employing particles formed using the P100-E15 polymer (Figure 1). PMs that were not covalently cross-linked dissociated at an SDS concentration of 5 mg/mL as seen by the rapid drop in size and huge increase in PDI. At concentrations above its CMC (1.7–2.3 mg/mL), SDS is known to solubilize amphiphilic block copolymers into a mixed micelle system resulting in destabilization of the PMs.^48^ In contrast, the cross-linked micelles maintained an almost constant PDI (<0.1) and gradual increase in size with increasing surfactant concentration. Notably, there is a proportional increase in Z-Ave from 47 to 87 nm of the cross-linked micelles with increasing SDS concentration, which is consistent with results observed previously for similar CCPMs and can likely be ascribed to the absorption of SDS by the micelles.^23^ This observation indicates that stable CCPMs were formed exploiting cross-linker **5**.

**Figure 2:**
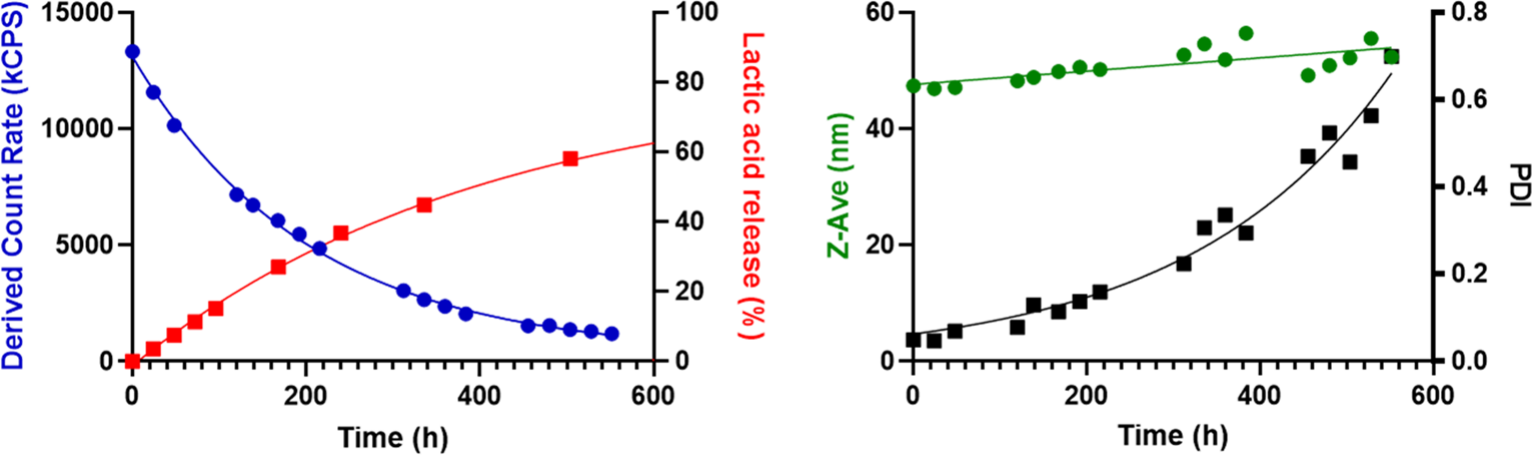
Degradation characteristics of P100-E15 based CCPMs under physiological conditions (pH 7.4, 37 °C) at a polymer concentration of 7.5 mg/mL. Derived count rate (blue circles), Z-Ave (green circles), and PDI (black squares) were determined by DLS measurements at 37 °C. Lactic acid formed % (red squares) was determined by UPLC.

Degradation characteristics of CCPMs based on P100-E15 were investigated by DLS measurements, in accordance with a previously published procedure.^16^Figure 2 shows an 90% decrease in light scattering intensity (SI) over a period of 480 h upon their incubation under physiological conditions (37 °C, pH 7.4). The diameter of the CCPMs increased from 47 to 56 nm, indicating swelling of the particles accompanied by a substantial increase in PDI. The degradation of the CCPMs is caused by the ester hydrolysis of lactate moieties in the free and cross-linked HPMAmLac_*n*_ side chains as has been previously reported.^49,50^ This also results in the release of lactic acid as shown in Figure 2, with a 60% of total lactic acid content release after 480 h (90% decrease in SI). Importantly, these results show that the CCPMs degrade under physiological conditions through the expected ester hydrolysis of lactate side chains and lactate esters in the cross-links, which is a crucial feature for *in vivo* applications.

### Dye Loading

To demonstrate the versatile options to covalently entrap model compounds using the trifunctional cross-linker, we loaded the CCPMs with two different dyes, Sulfo.Cy5 and BODIPY, employing two different copper-free click chemistry handles (dibenzocyclooctyne (DBCO) and 3,3,6,6-tetramethylthiacycloheptyne (TMTHSI), respectively). A third ring strained alkyne, bicyclo[6.1.0]non-4-yn-9-ylmethanol (BCN) was employed to model the click reaction and gain sufficient quantities for NMR and IR analysis.

Since ring strained alkyne functionalities such as BCN and DBCO are known to react with free thiols present on cysteines,^51,52^ the dyes need to be clicked to the cross-linker in the oxidized disulfide form. We therefore coupled BCN-OH in slight excess with the azide and performed NMR and IR to confirm that azide–alkyne coupling rather than thiol–alkyne formation occurred (Figure S6.1). Indeed, an upshift of the cross-linker protons neighboring the azide was observed in the NMR spectra and a depletion of the azide signal at 2100 cm^–1^ in the IR spectra confirmed the formation of a triazole ring following the azide–alkyne click reaction. Furthermore, the identity of the coupled product was confirmed by mass spectrometry (Figure S2.2).

Both Sulfo.Cy5-DBCO as well as BODIPY-TMTHSI can react with azides by strain-promoted azide alkyne cyclo-addition reaction. After the click reaction, the disulfides were reduced using TCEP to render the dye-cross-linker conjugates ready for the NCL cross-linking reaction, as confirmed by MALDI-MS (Figures S2.3 and S2.4). Both the clicking of the dyes as well as opening of the disulfide bridges was performed in a simple one pot fashion.

The successful entrapment of either Sulfo.Cy5-DBCO or BODIPY-TMTHSI into the CCPMs was shown by HPLC of samples before purification (Figures S3.3 and S3.4). Entrapment was analyzed on the CCPMs as such since this approach of dye entrapment is nonreversible. A significant increase in absorbance at 650 nm was found for Sulfo.Cy5 and BODIPY entrapped CCPMs compared to empty CCPMs accompanied by the depletion of the free dye signal, indicating successful entrapment with >95% efficiency. DLS measurements of the purified CCPMs using a fluorescence filter setup found a Z-Ave of 47 and 52 nm with a PDI of 0.04 and 0.14 for the S.Cy5 and BODIPY loaded CCPMs respectively, showing that dye cargo incorporation did not significantly alter the CCPM size.

### Cell Viability and Internalization

To demonstrate the cytocompatibility of our dye loaded CCPMs and their possible cellular internalization,^53^ we performed *in vitro* cell studies using the BODIPY-TMTHSI formulation. Prostate cancer cells (PC3), which previously have shown high uptake of similar CCPMs,^44^ were cultured to visualize uptake of the BODIPY loaded CCPMs. These CCPMs had a 2% dye weight ratio relative to the polymers and were incubated for 24 h at a polymer concentration of 0.1 mg/mL with the cells.

**Figure 3:**
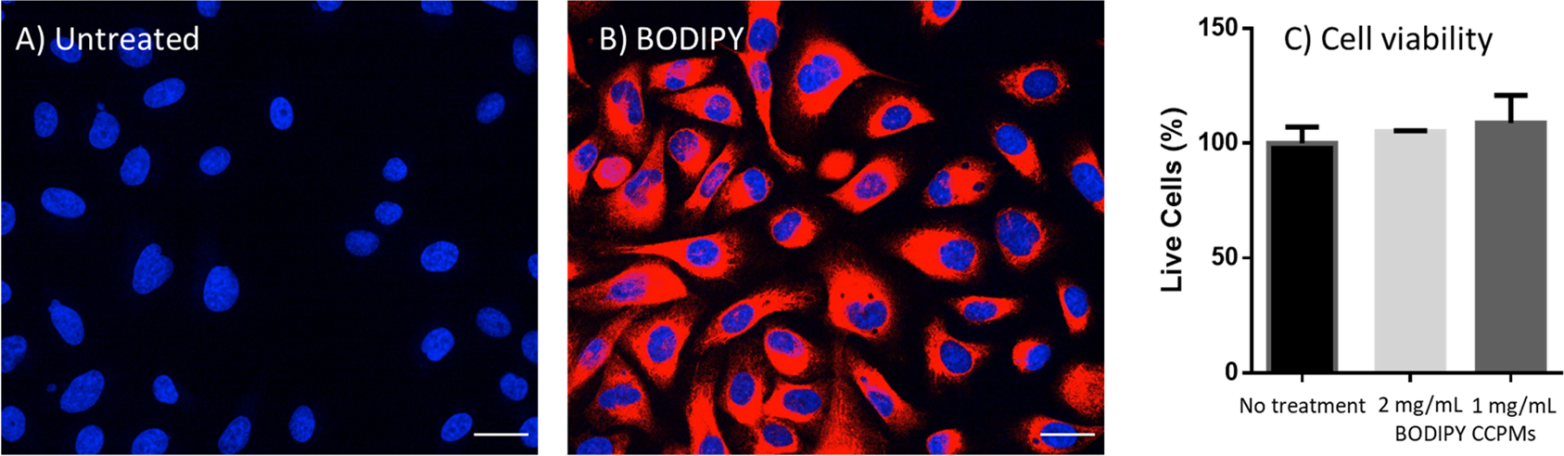
Confocal laser scanning microscopy images of PC3 cells incubated for 24 h with empty CCPMs (A) or BODIPY loaded CCPMs (B). Cells were treated with 2 μg/mL Hoechst nuclei stain (blue signal) prior to microscopy. Scale bar: 30 μm. Cell viability was determined in triplicate by MTS staining for BODIPY loaded CCPMs (C).

Significant uptake of the CCPMs was observed for BODIPY entrapped CCPMs as shown by the laser confocal scanning microscopy imaging (Figure 3A,B). Uptake was further confirmed by cell associated fluorescence flow cytometry measurements (Figure S7.1), which is in line with previously published results employing a similar PEGylated CCPM system.^44^

Importantly, no cytotoxicity on the PC3 cells up to at least a CCPM concentration of 2 mg/mL (20-fold higher than the uptake study) was observed, indicating a good cytocompatibility (Figure 3 C).

## Conclusion

New generations of pharmaceuticals are increasingly becoming biological and peptide based in nature, increasing their fragility toward chemistries such as free radical reactions. The entrapment of these fragile compounds in nanoparticles for extended circulation and tumor accumulation is of interest not only for therapeutic efficiency and safety but also cost reduction, for which orthogonal chemistries need to be developed. We developed a physiologically degradable CCPM platform stabilized by mild, biorthogonal NCL cross-linking with a clickable azide handles for potential (pro)drug conjugation. By using the fluorescent dyes Sulfo.Cy5 and BODIPY as model compounds, we demonstrated successful entrapment and cytocompatibility of these CCPMs. Clearly, for the entrapment of active pharmaceutical compounds and subsequent (triggered) release of active pharmaceutical compounds, successful linker (prodrug) design is paramount and future work will be focused toward this avenue. The platform described here serves as a steppingstone for the orthogonal entrapment of therapeutics into CCPMs and to provide manifold opportunities toward imaging and therapy.

## Supporting Information Available

^1^H NMR spectra of compounds **1**–**5**, polymer P100, ETSA, and polymer P100E15 (Figures S1.1–1.11); mass spectra of cross-linker, cross-linker coupled to BCN-OH, cross-linker coupled to Suflo.Cy5-DBCO, and cross-linker coupled to BODIPY-TMTHSI (Figures S2.1–2.4); HPLC chromatograms of P100E15 and ETSA, CCPMs before and after purification, CCPMs loaded with Sulfo.Cy5, and CCPMs loaded with BODIPY (Figures S3.1–3.4); UHPLC chromatograms of NaOH hydrolyzed P100E15 for ETSA and total polymer content determination (Figures S4.1 and S4.2); size exclusion chromatogram of purified empty CCPMs (Figure S5.1); schematic representation of azide selective cross-linker reaction with BCN-OH showing ^1^H NMR and IR data (Figure S6.1); cell associated fluorescence of PC3 cells incubated with BODIPY loaded CCPMs (Figure S7.1) (PDF)

## List of Abbreviations

ABCPA: 4,4-azobis(4-cyanopentanoic acid)
ACN: acetonitrile
BCN: bicyclo[6.1.0]nonyne
BODIPY: 4,4-difluoro-4-bora-3a,4a-diaza-*s*-indacene
CCPM: core-cross-linked polymeric micelle
CMC: critical micellization concentration
CP: cloud point
CMT: critical micellization temperature
DCC: *N*,*N*′-dicyclohexylcarbodiimide
DCM: dichloromethane
DCR: derived count rate
DCU: dicyclohexylurea
DLS: dynamic light scattering
DMF: dimethylformamide
DMSO: dimethyl sulfoxide
DPTS: *N,N*-dimethylaminopyridinium *p*-toluenesulfonate
ETSA: ethylthioglycolate-succinic acid
ESI: electrospray ionization
FBS: fetal bovine serum
GPC: gel permeation chromatography
HATU: hexafluorophosphate azabenzotriazole tetramethyl uronium
HPMAmLac_*n*_: *N*-2-hydroxypropyl methacrylamide mono/di lactate
HPLC: high pressure liquid chromatography
IR: Infrared
MALDI: matrix assisted laser desorption ionization
mPEG: methoxy polyethylene glycol
MPS: mononuclear phagocyte system
MS: mass spectrometry
MTS: 3-(4,5-dimethylthiazol-2-yl)-5-(3-carboxymethoxyphenyl)-2-(4-sulfophenyl)-2H-tetrazolium
NCL: native chemical ligation
PDI: polydispersity index
PEG: polyethylene glycol
PM: polymeric micelle
Pn: mPEG_5000_-*b*-P(HPMAmLac_1_-*co*-HPMAmLac_2_) copolymer
Pn-En: mPEG_5000_-*b*-P(HPMAmLac_*n*_-*co*-HPMAmLac_*n*_-ETSA) copolymer
PTFE: polytetrafluoroethylene
SDS: sodium dodecyl sulfate
Sulfo.Cy5: sulfonated cyanine 5
TCEP: Tris(2-carboxyethyl)phosphine
TFA: trifluoroacetic acid
TMTHSI: 3,3,6,6-tetramethylthiacycloheptyne sulfoximine
UHPLC: ultra high pressure liquid chromatography
