# Supplementary material for: Orthogonal Covalent Entrapment of Cargo into Biodegradable Polymeric Micelles via Native Chemical Ligation
Source: Biomacromolecules. 2022 Aug 31;24(10):4385–96. doi: 10.1021/acs.biomac.2c00865 (PMC10565831; doi:10.1021/acs.biomac.2c00865)
Supplement: Supplementary file 1 — bm2c00865_si_001.pdf [file bm2c00865_si_001.pdf]

Supporting Information (SI)

The orthogonal covalent entrapment of cargo into biodegradable polymeric micelles *via* native chemical ligation

Erik R. Hebels[1*], Felix Bindt[1], Johanna Walther[1], Michiel van Geijn[2], Jimmy Weterings[2], Qizhi Hu[2], Claudio Colombo[2], Rob Liskamp[2], Cristianne Rijcken[2], Wim E. Hennink[1] and Tina Vermonden[1*]

[1] Division of Pharmaceutics, Utrecht Institute for Pharmaceutical Sciences (UIPS), Utrecht University, 3508 TB Utrecht, the Netherlands.

[2] Cristal Therapeutics, 6229 EV Maastricht, the Netherlands

[*] Corresponding Authors

1. NMR Spectra

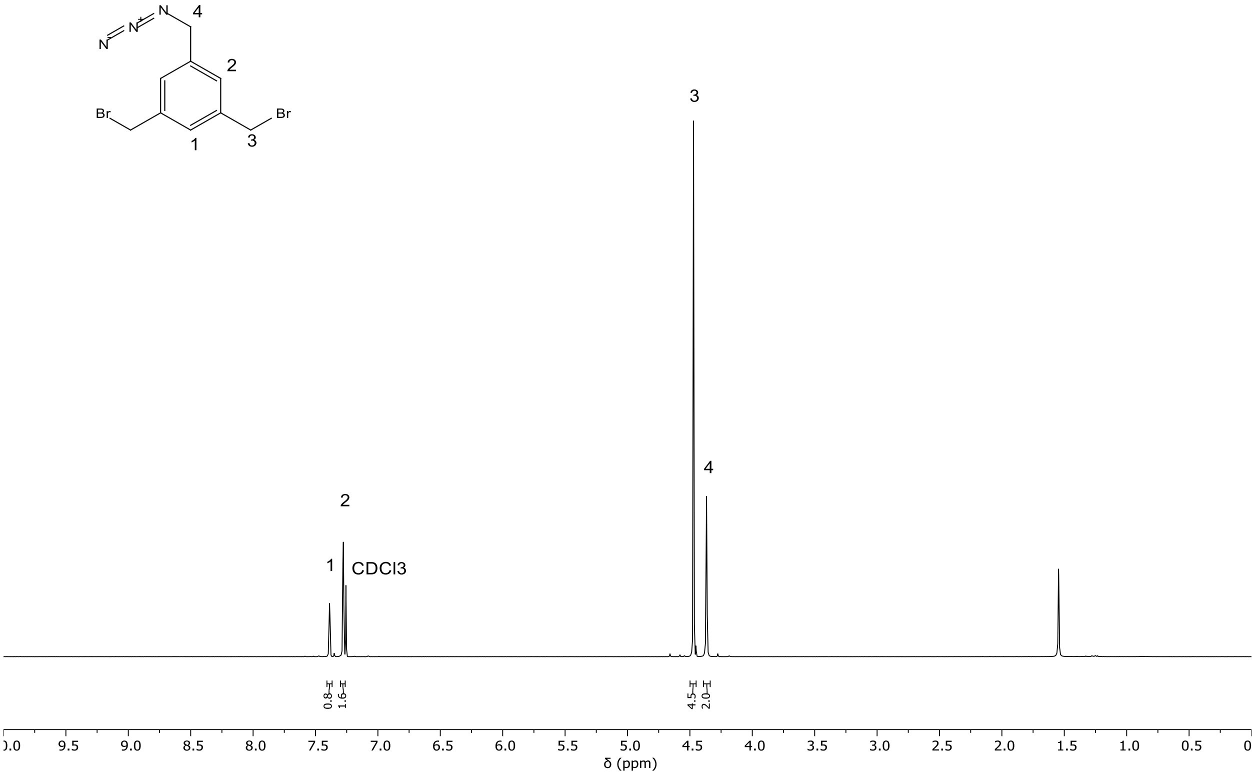

Figure S1.1: ^{1}H NMR spectrum of compound ***1*** *(1-(azidomethyl)-3,5-bis(bromomethyl)benzene). The solvent was$CDCl_3$.*

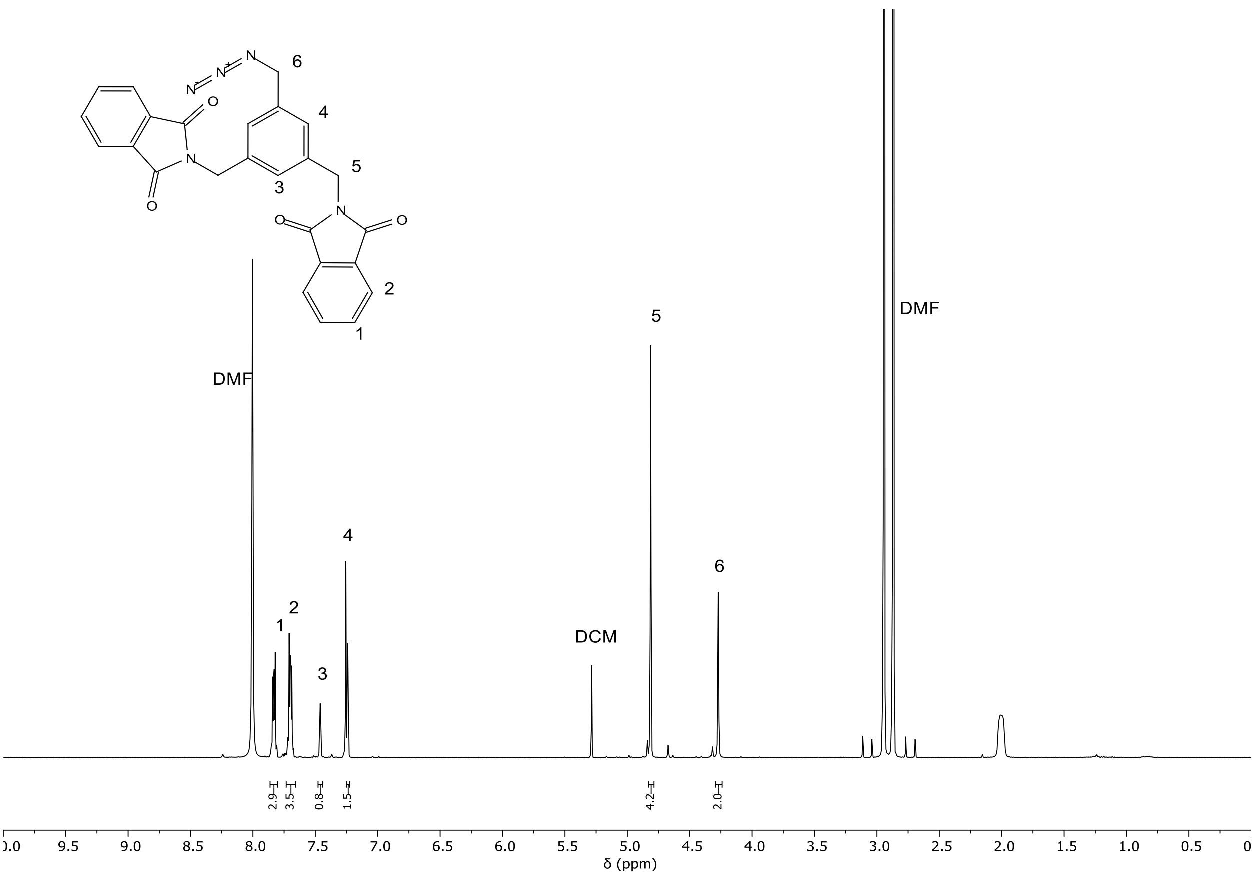

*Figure S1.2: ^{1}H NMR spectrum of compound **2** (1-(azidomethyl)-3,5-bis(phthalamidomethyl)benzene). The solvent was $CDCl_3$.*

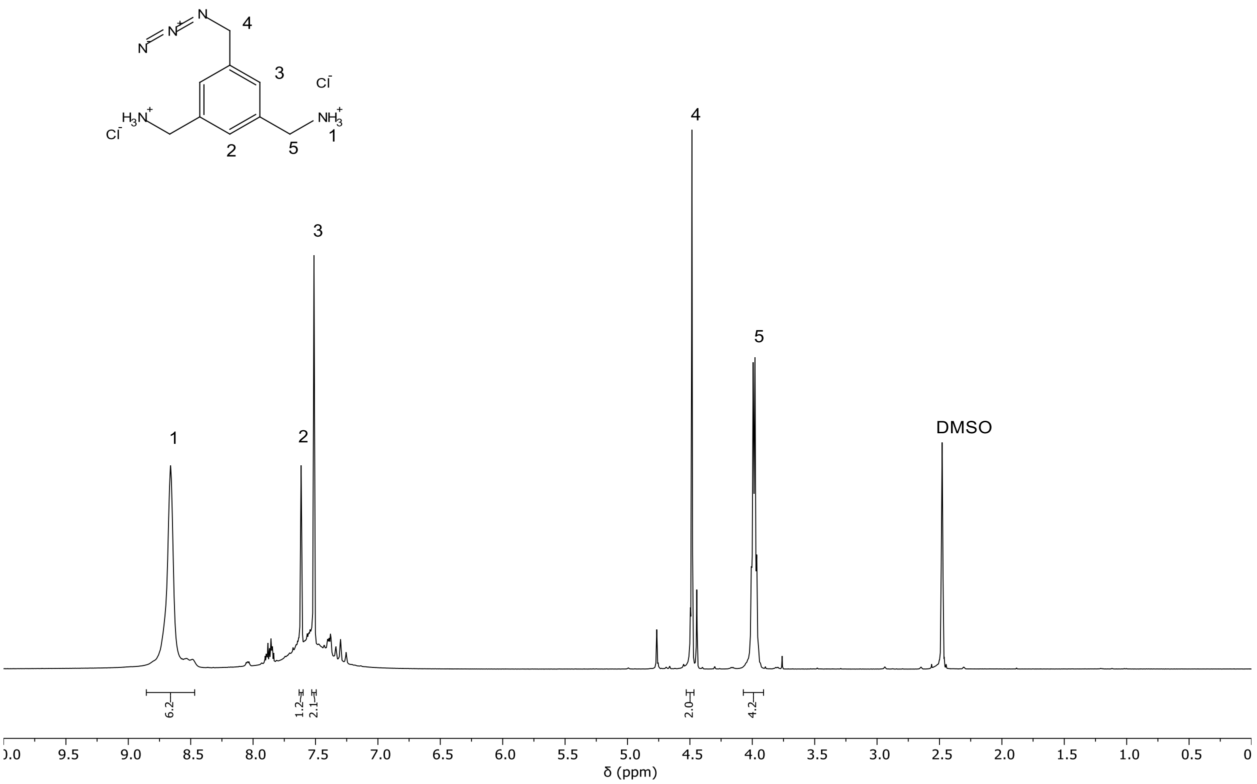

*Figure S1.3: ^{1}H NMR spectrum of compound **3** (1-(azidomethyl)-3,5-bis(aminomethyl)benzene). The solvent was deuterated DMSO.*

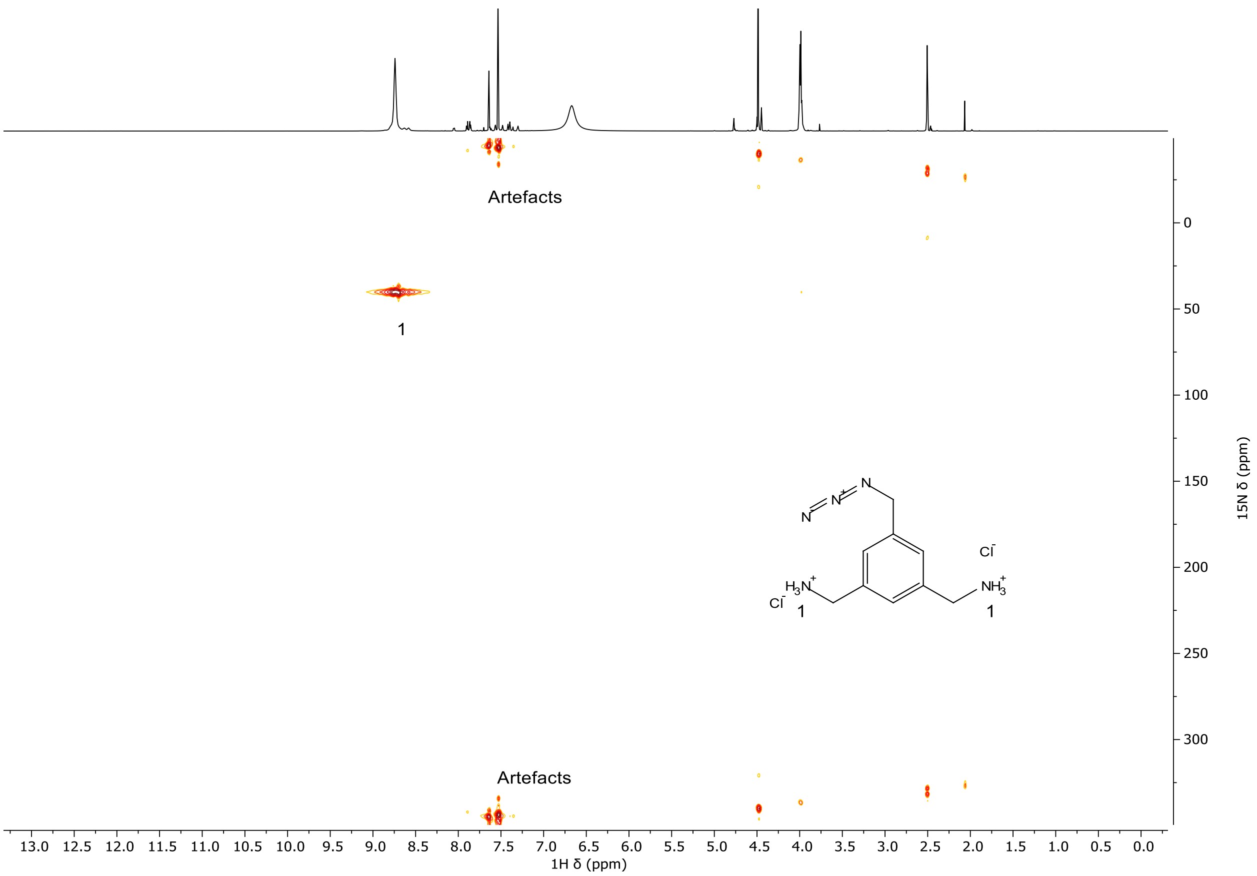

*Figure S1.4: ^{15}N HSQC NMR spectrum of compound **3**. The solvent was deuterated DMSO.*

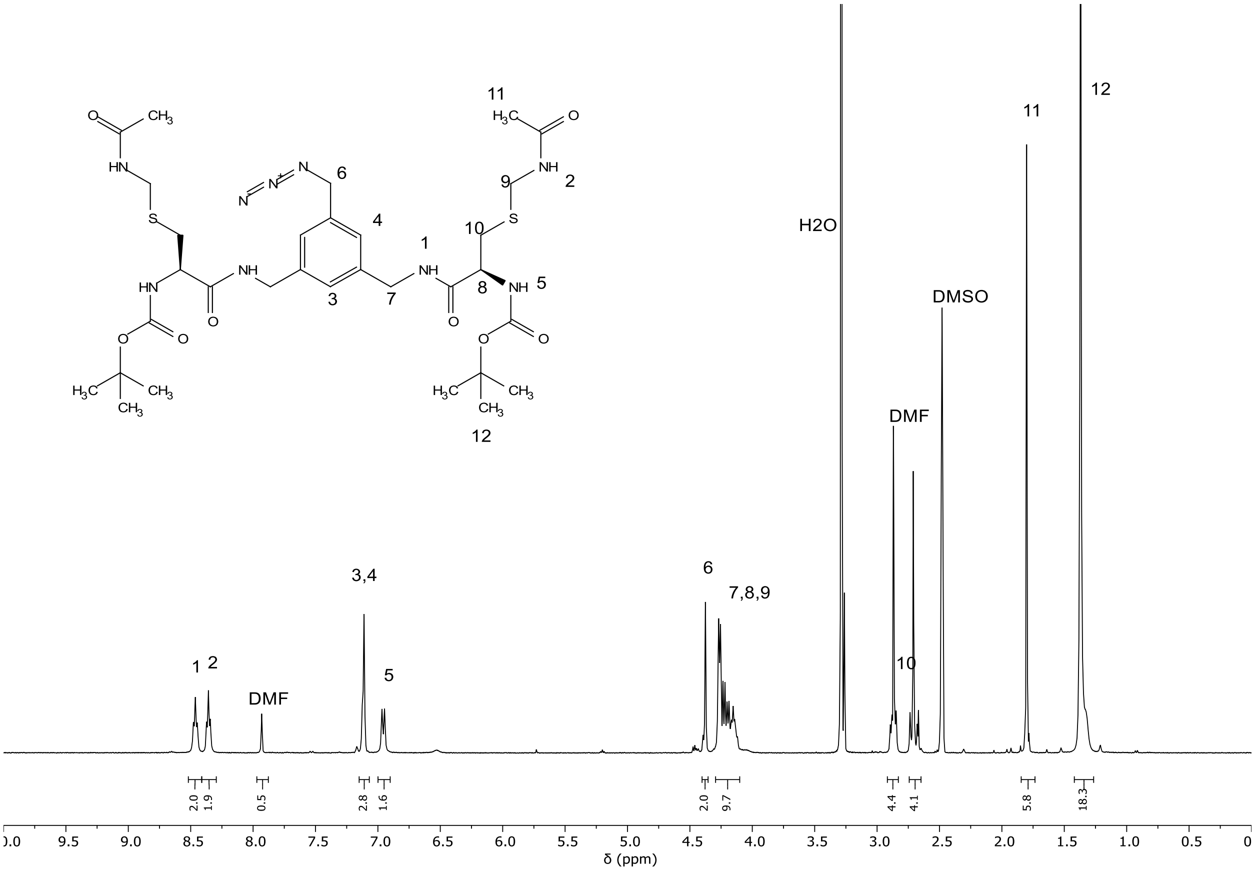

Figure S1.5: ^{1}*H NMR spectrum of compound* ***4****. The solvent was deuterated DMSO.*

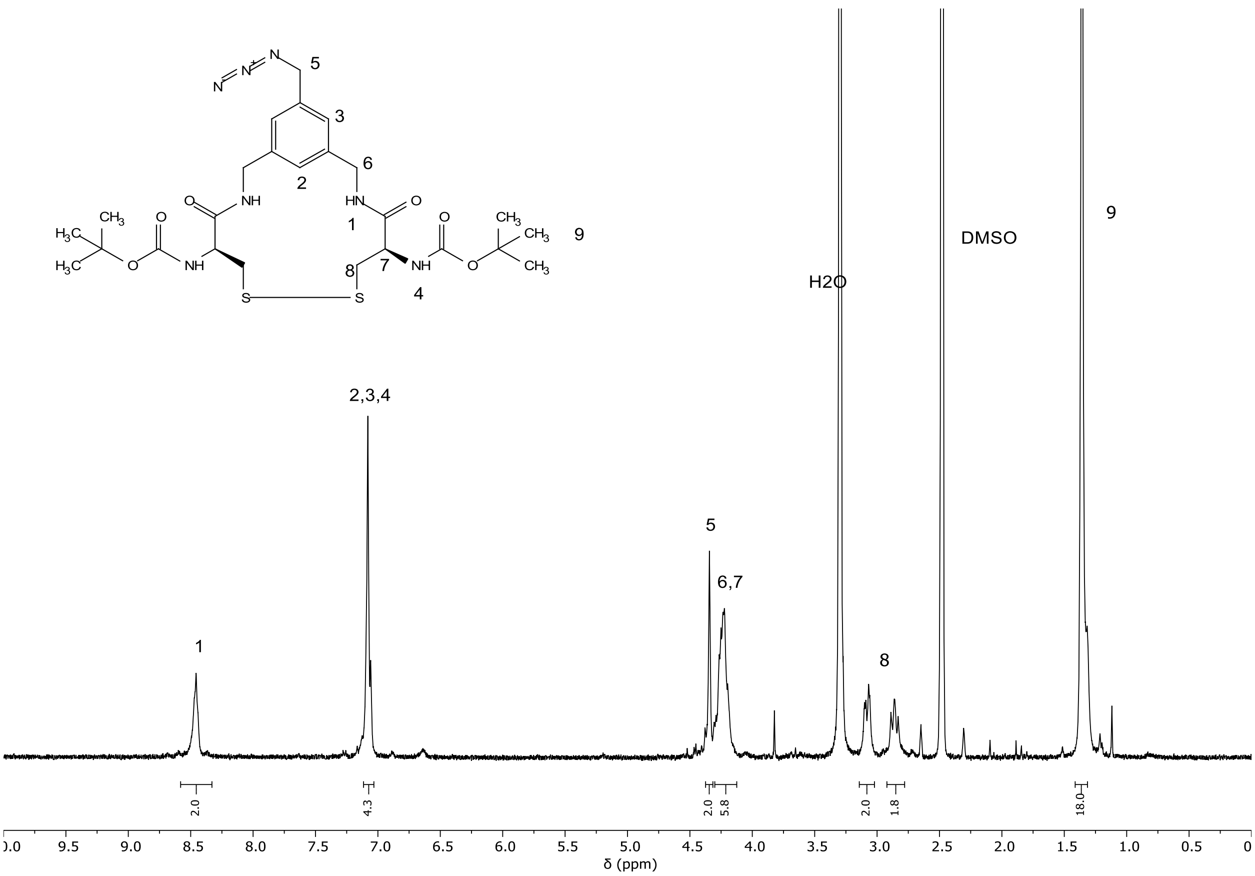

*Figure S1.6: ^{1}H NMR spectrum of Acm deprotected compound **4**. The solvent was deuterated DMSO.*

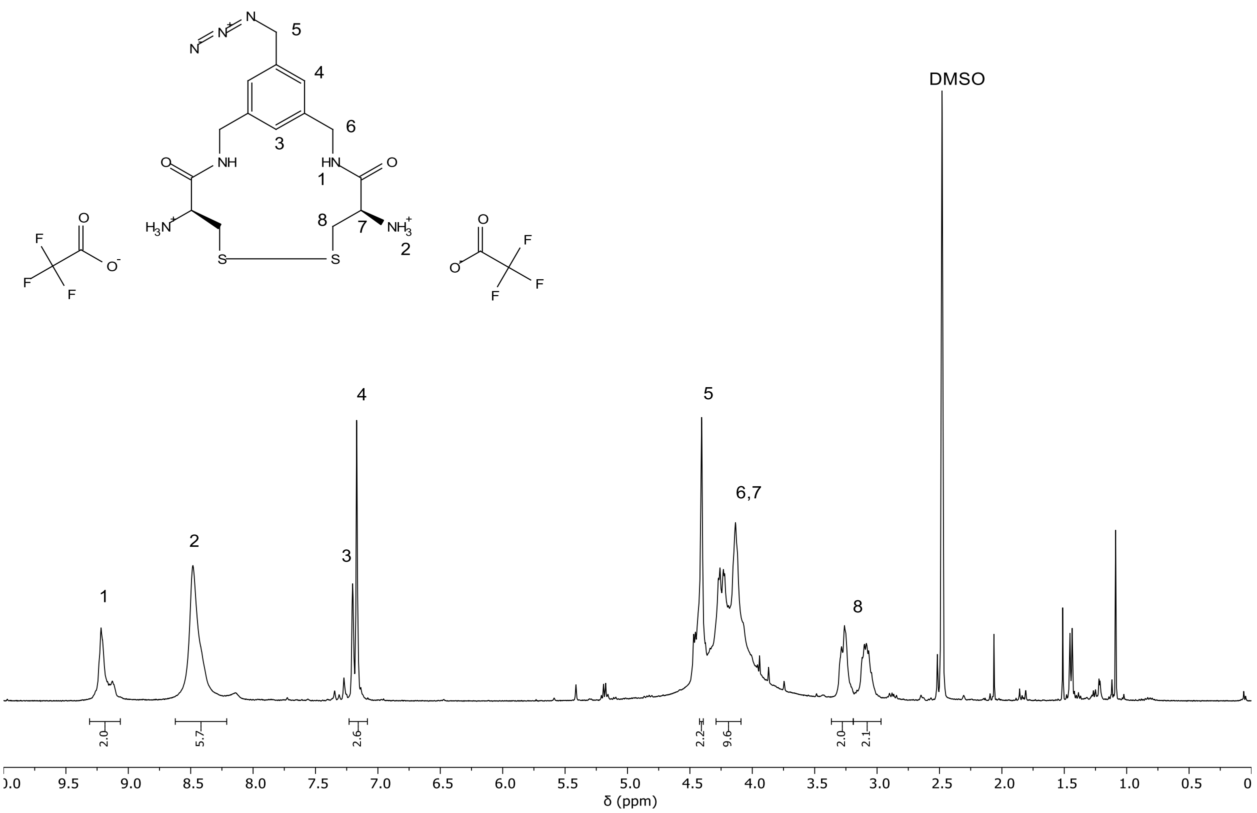

*Figure S1.7: ^{1}H NMR spectrum of compound **5**. The solvent was deuterated DMSO.*

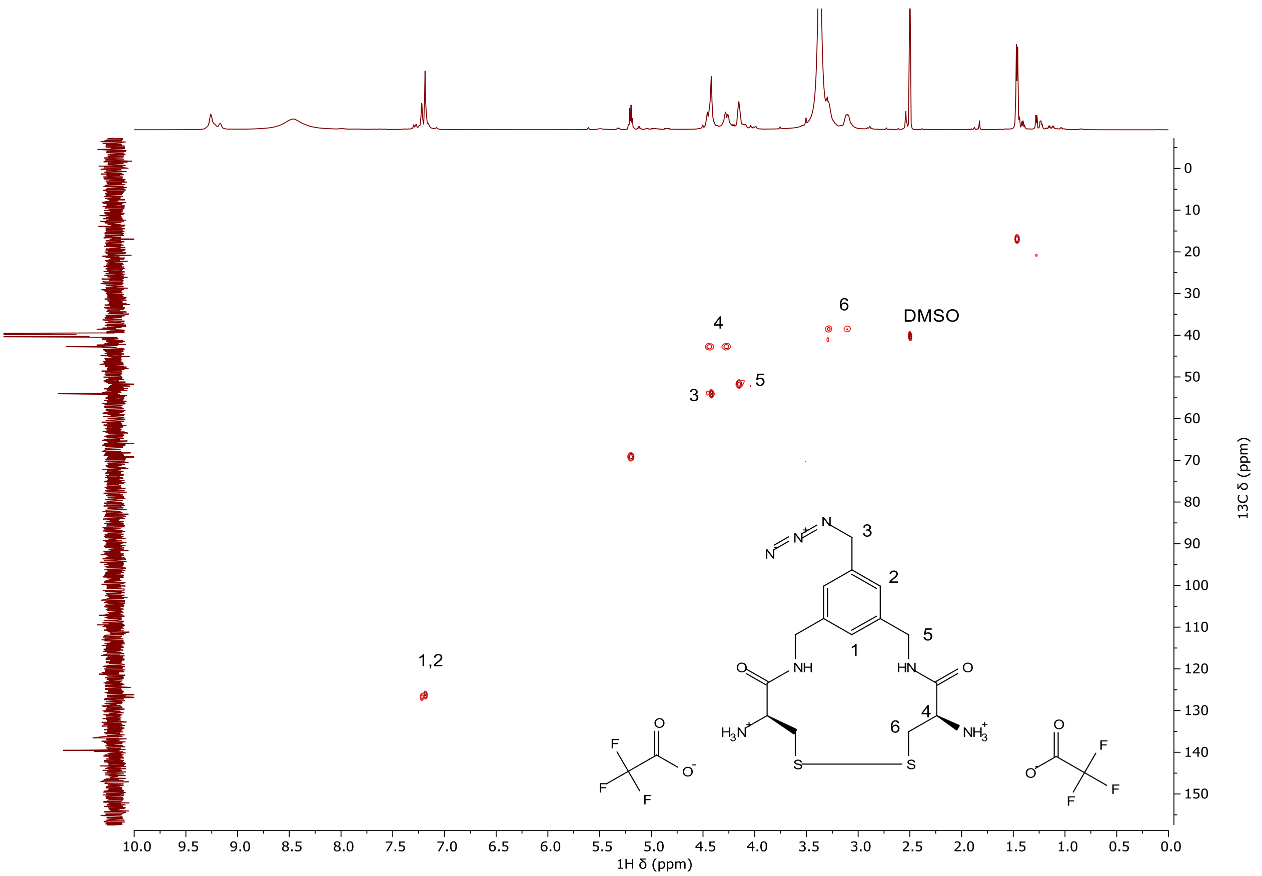

*Figure S1.8: HSQC NMR spectrum of compound **5**. The solvent was deuterated DMSO with a molecular sieve after rotary evaporation with toluene.*

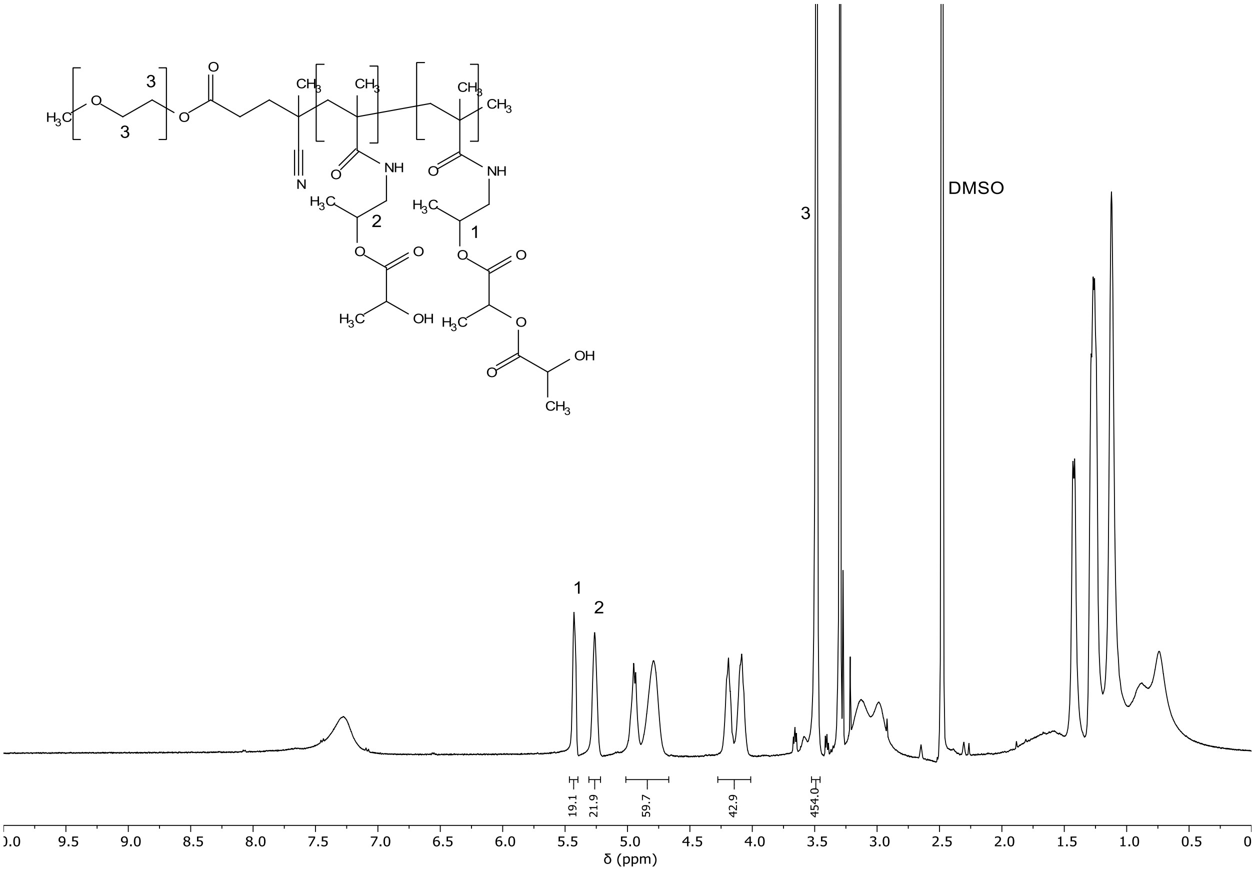

Figure S1.9: ^{1}H NMR spectrum of polymer ***P100*** *(PEG$_{5000}$-b-P(HPMAmLac$_1$-co-HPMAmLac$_2$)). The solvent was deuterated DMSO.*

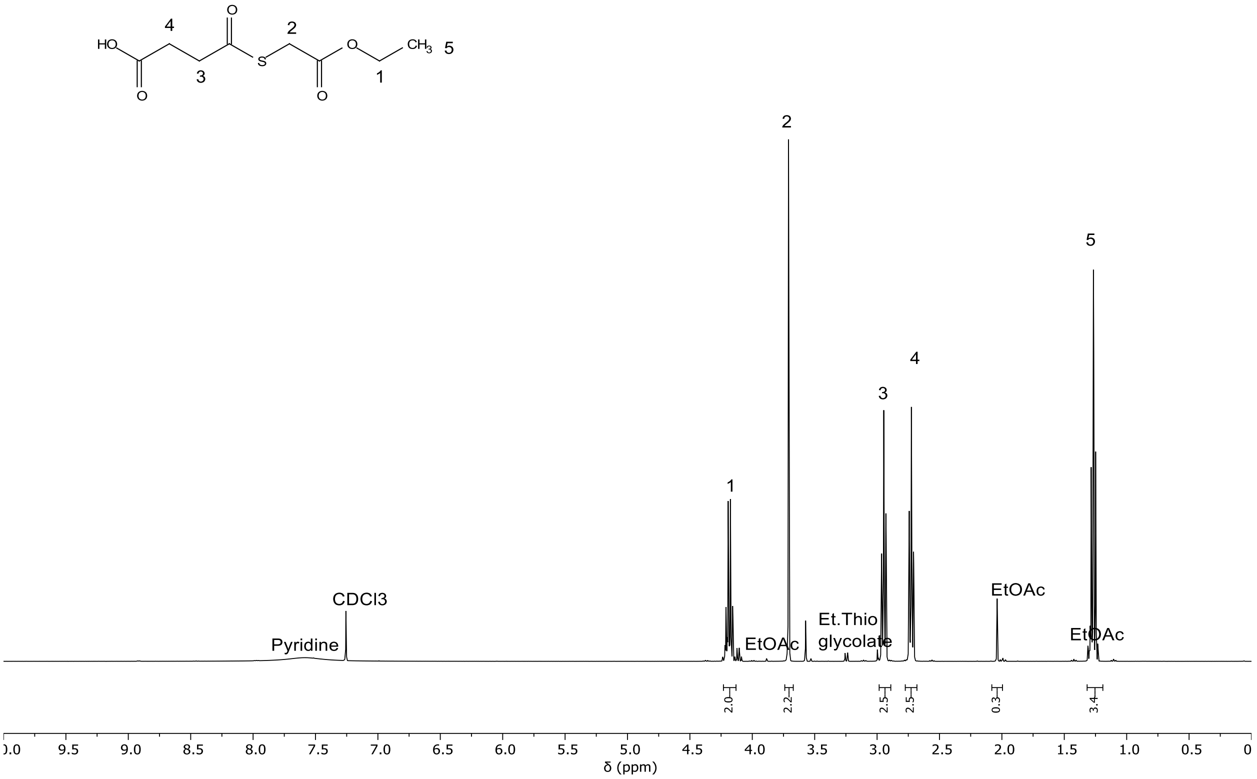

Figure S1.10: ^{1}H NMR spectrum of Ethyl Thioglycolate-Succinic Acid (ETSA). The solvent was $CDCl_3$.

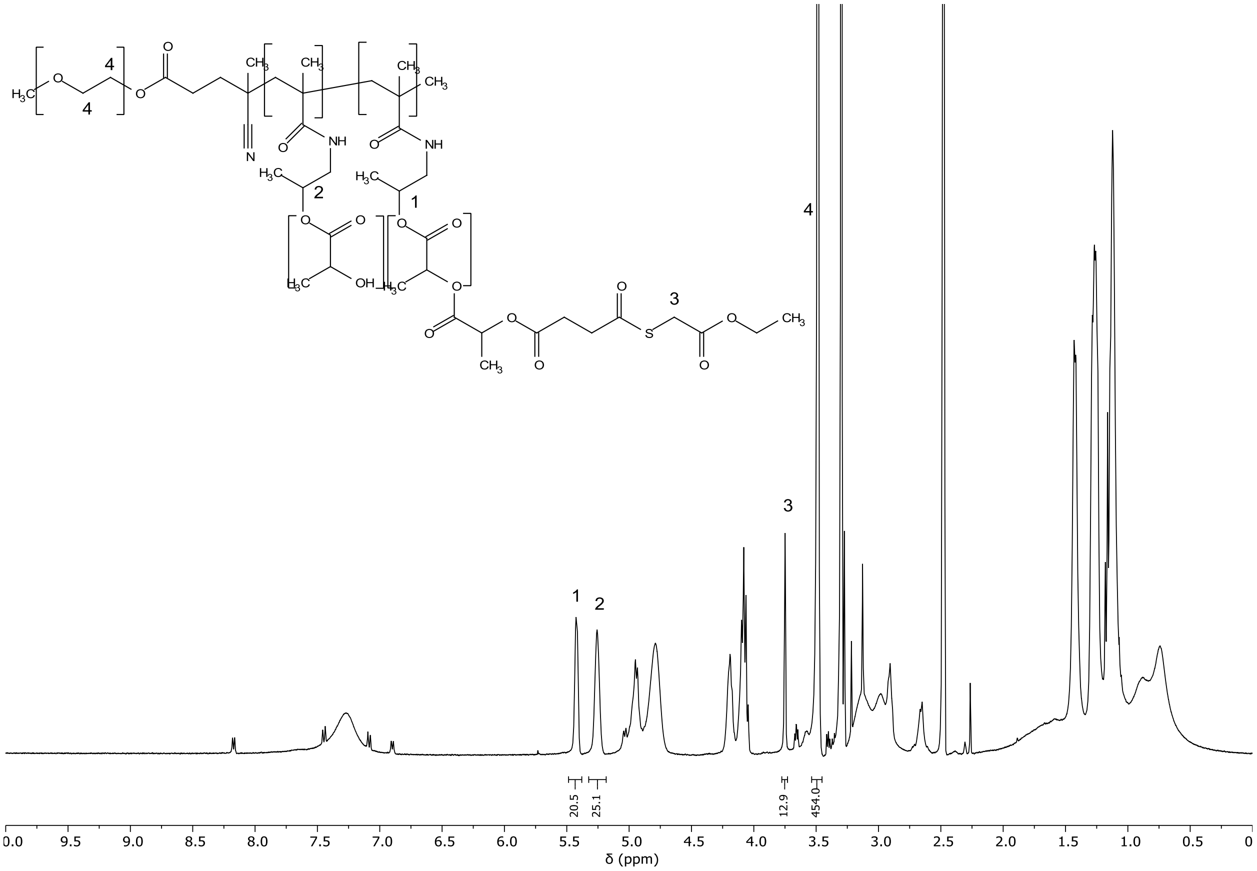

*Figure S1.11: ^{1}H NMR spectrum of polymer **P100-E15** (PEG$_{5000}$-b-P(HPMAmLac$_{n}$-co-HPMAmLac$_{n}$-ETSA)). The solvent was deuterated DMSO. Expected integral for coupled ETSA based on feed of **P100-E15** was 12.0, measured 12.9*

2. Mass Spectra

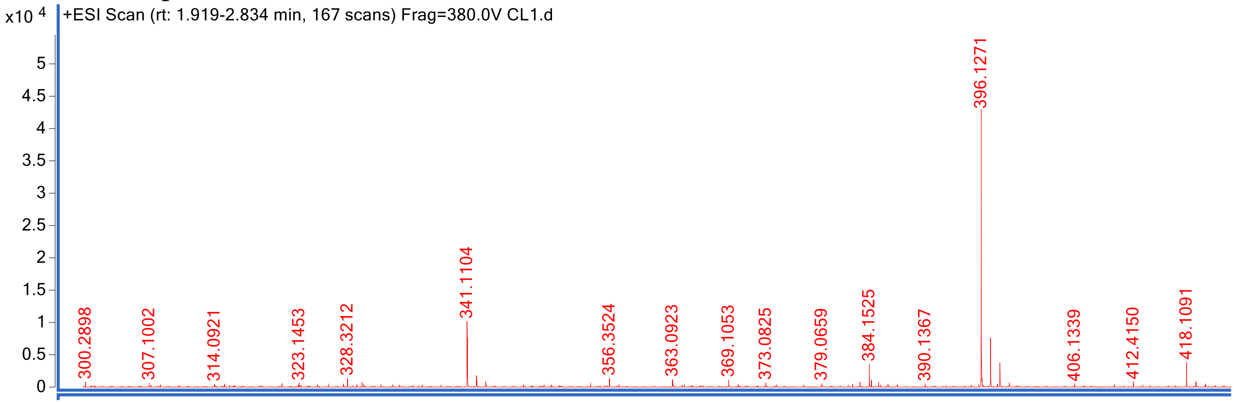

Figure S2.1: HR-MS spectrum of the trifunctional crosslinker, compound 5. Expected mass for $C_{15}H_{22}N_7O_2S_2^+$ is 396.1271, measured 396.1271.

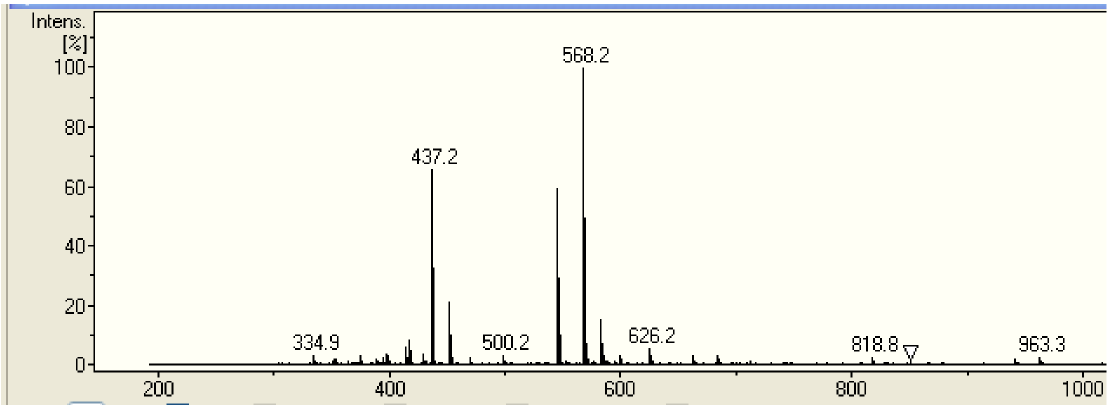

Figure S2.2: ESI-MS spectrum of the trifunctional crosslinker, compound 5 coupled with BCN-OH. Expected mass for $C_{25}H_{35}N_7O_3S_2^+$ is 546.2 and $C_{25}H_{35}N_7O_3S_2Na^+$ is 568.2, measured 568.2.

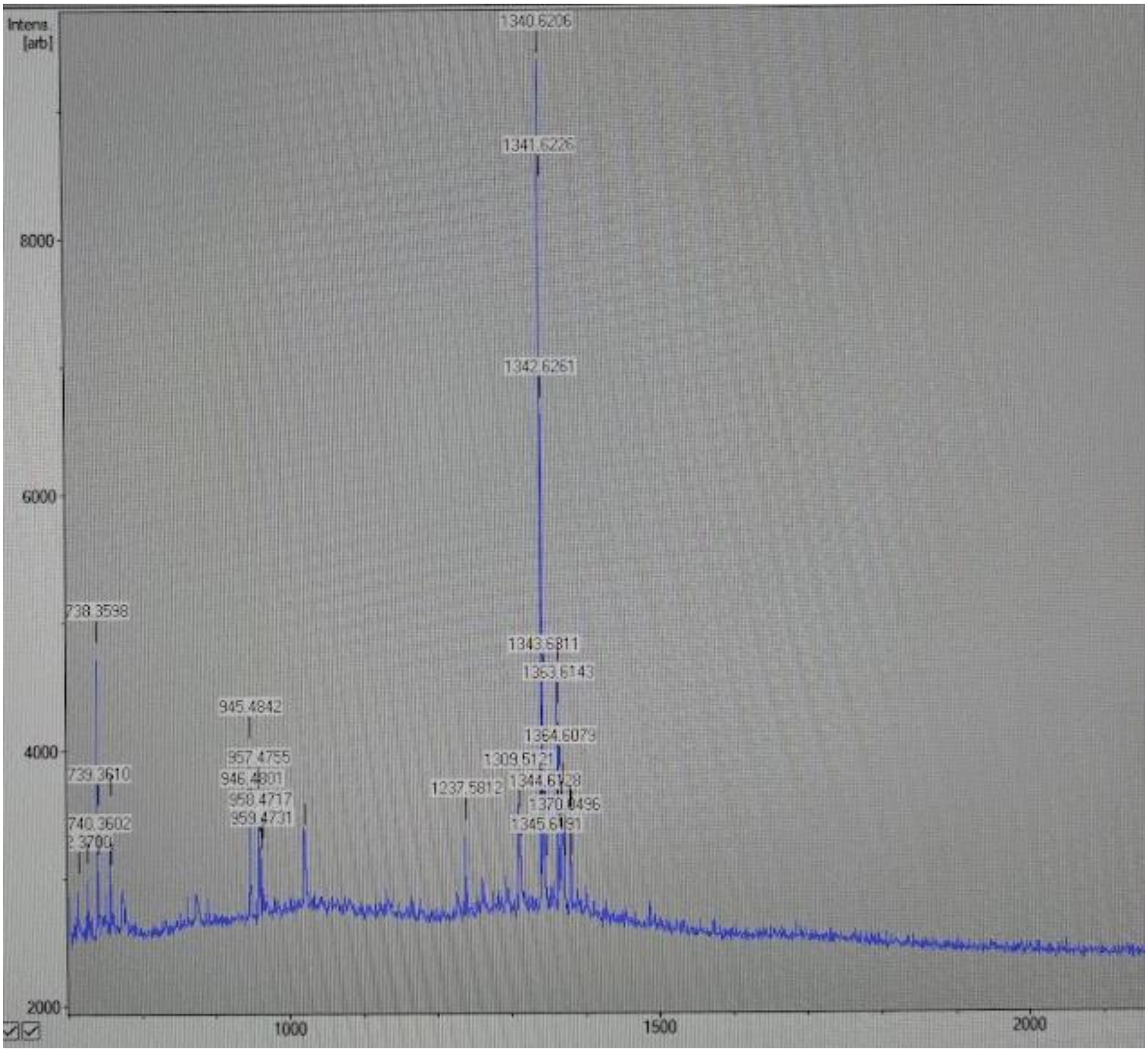

Figure S2.3: MALDI-MS spectrum of the trifunctional crosslinker, compound 5 coupled with Sulfo.Cy5-DBCO after TCEP reduction. Expected mass for $C_{68}H_{82}N_{11}O_{10}S_4^+$ is 1340.5, measured 1340.6.

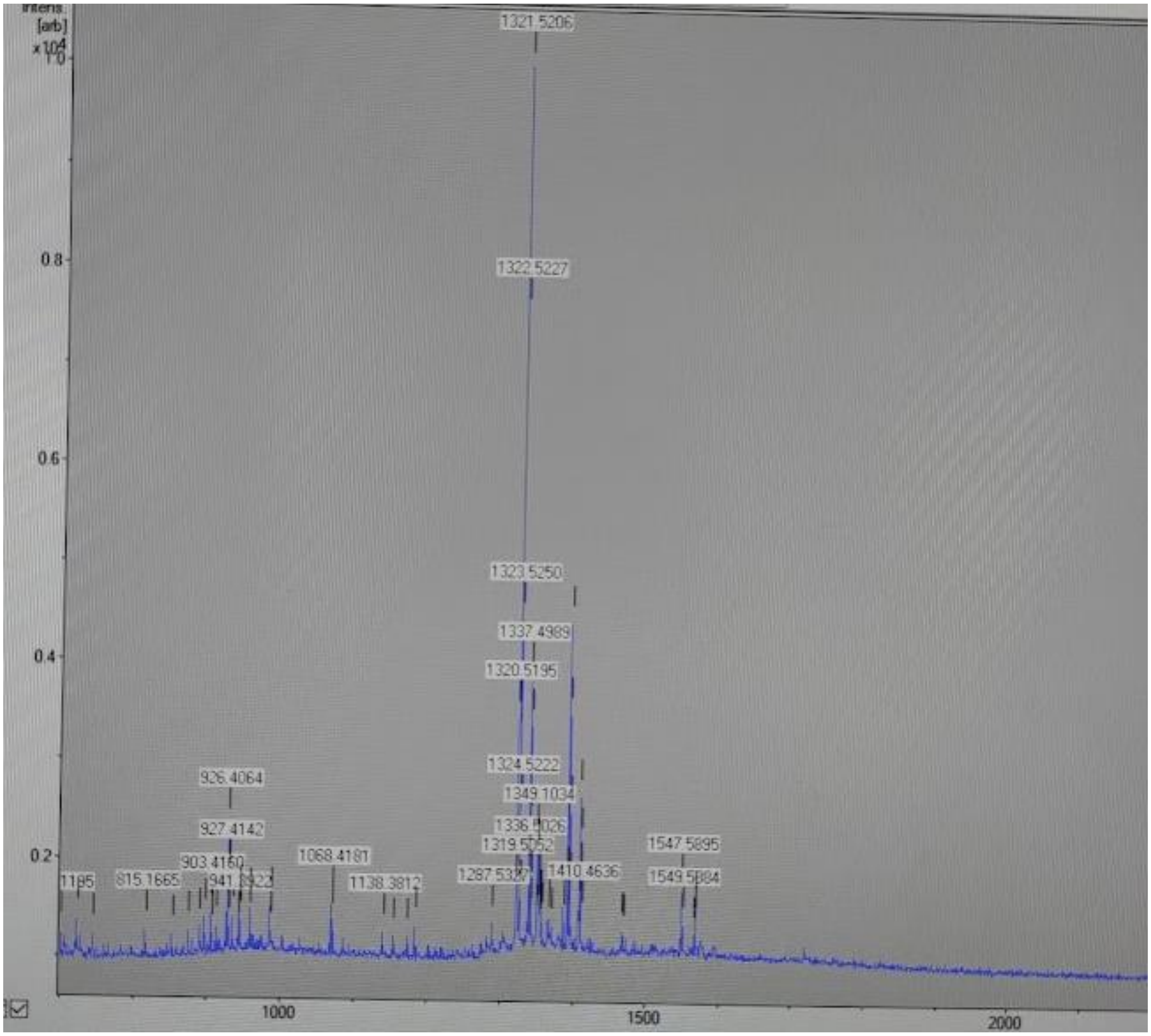

Figure S2.4: MALDI-MS spectrum of the trifunctional crosslinker, compound 5 coupled with BODIPY-TMTHSI after TCEP reduction. Expected mass for $C_{61}H_{82}N_{14}O_9S_3BF_2^+$ is 1299.6 and $C_{61}H_{81}N_{14}O_9S_3BF_2Na^+$ is 1321.5, measured 1321.5.

3. HPLC Chromatograms

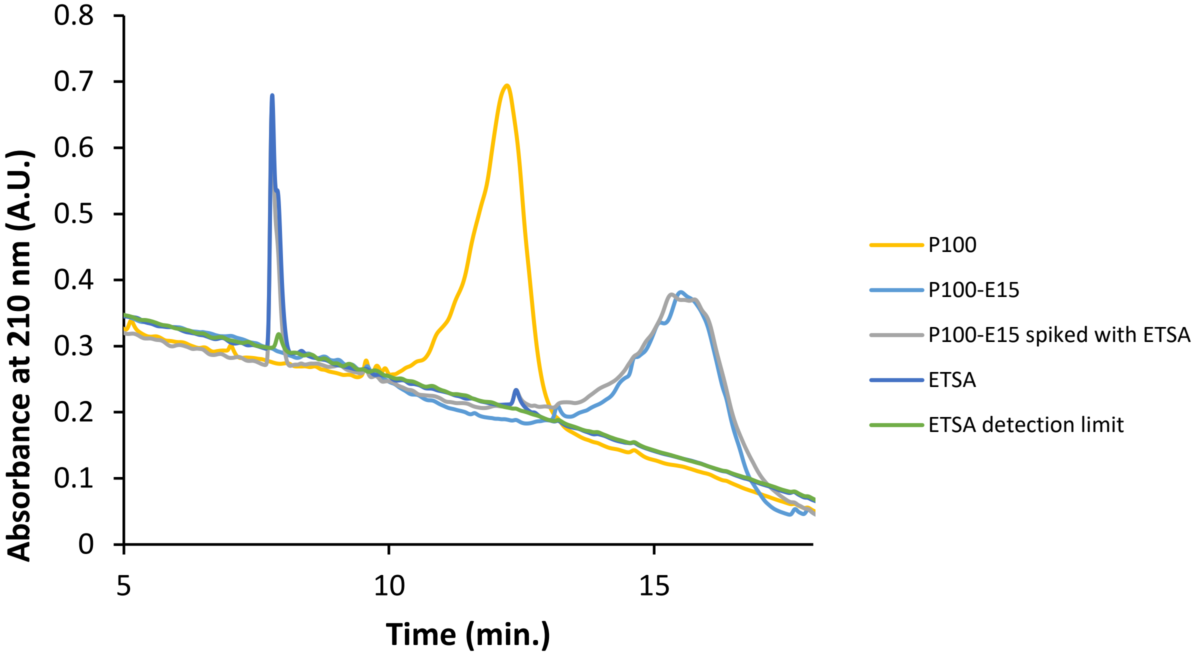

Figure S3.1: HPLC chromatograms of lead mPEG$_{5000}$-b-P(HPMAmLac$_1$-co-HPMAmLac$_2$) polymer before (yellow) and after (light blue) ETSA (blue) functionalization, including a spiked sample (grey) and 60 µg/mL ETSA (green) injection marking the detection limit. Injected at 10 mg/mL polymer concentration and 1 mg/mL ETSA concentration.

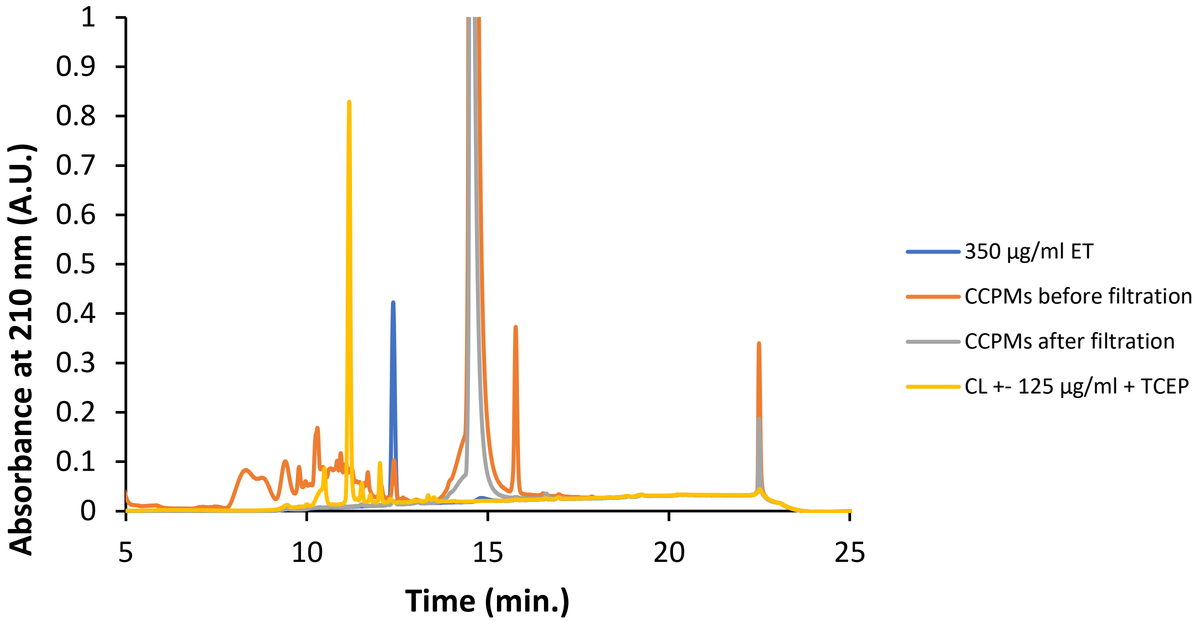

Figure S3.2: HPLC chromatograms of CCPMs before (orange) and after (grey) purification as well as ethyl thioglycolate (blue) and crosslinker treated with TCEP (yellow) references.

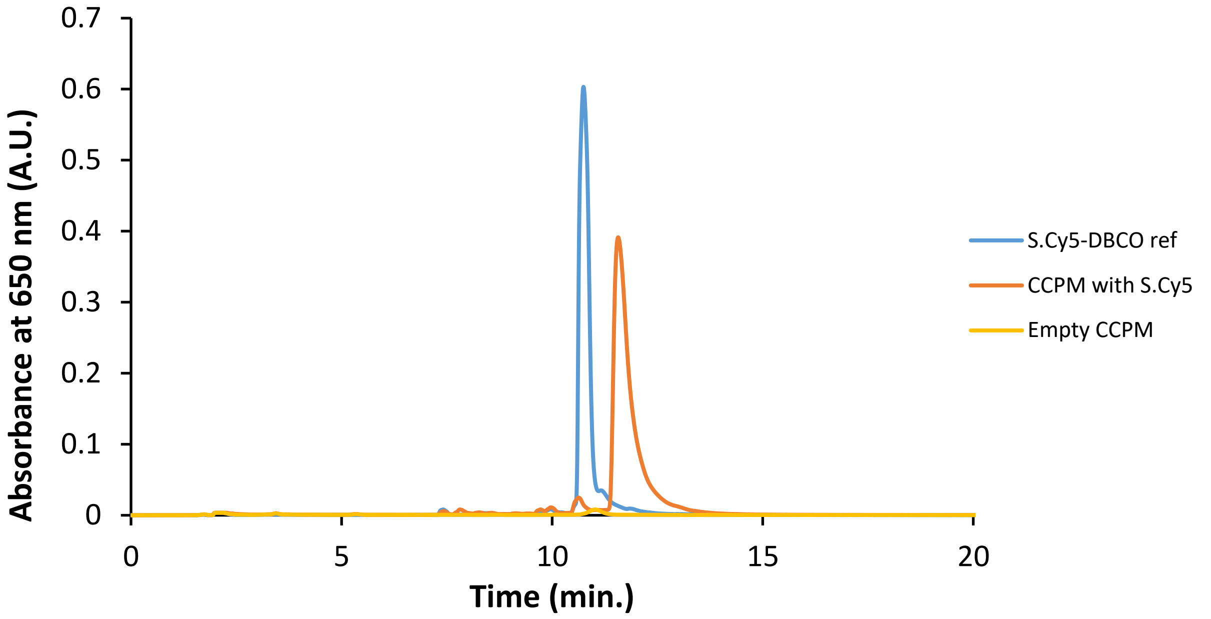

Figure S3.3: HPLC chromatograms of empty CCPMs (yellow), Sulfo-Cy5 loaded CCPMs (orange) and free Sulfo-Cy5-DBCO (blue). Samples taken before size exclusion purification of micelles.

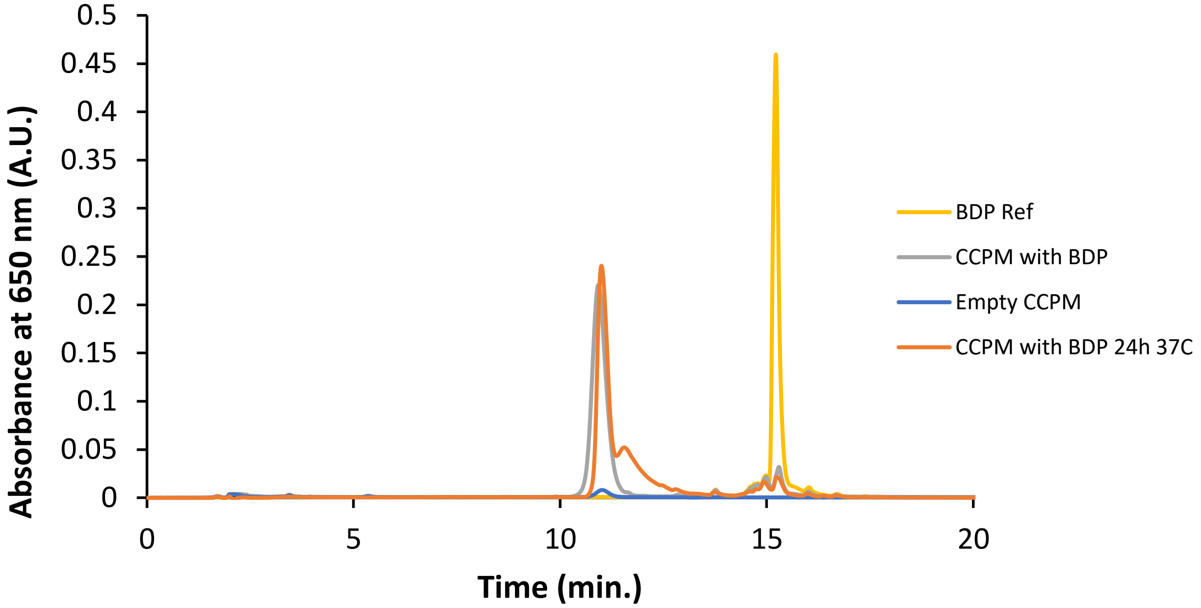

Figure S3.4: HPLC chromatograms of empty CCPMs (blue), BODIPY loaded CCPMs (grey) and free BODIPY-TMTHSI (yellow). Samples taken before size exclusion purification of micelles. After purification, the BODIPY loaded CCPMs were incubated at 37°C (orange) to confirm no undesired dye release occurred.

4. UHPLC Chromatograms

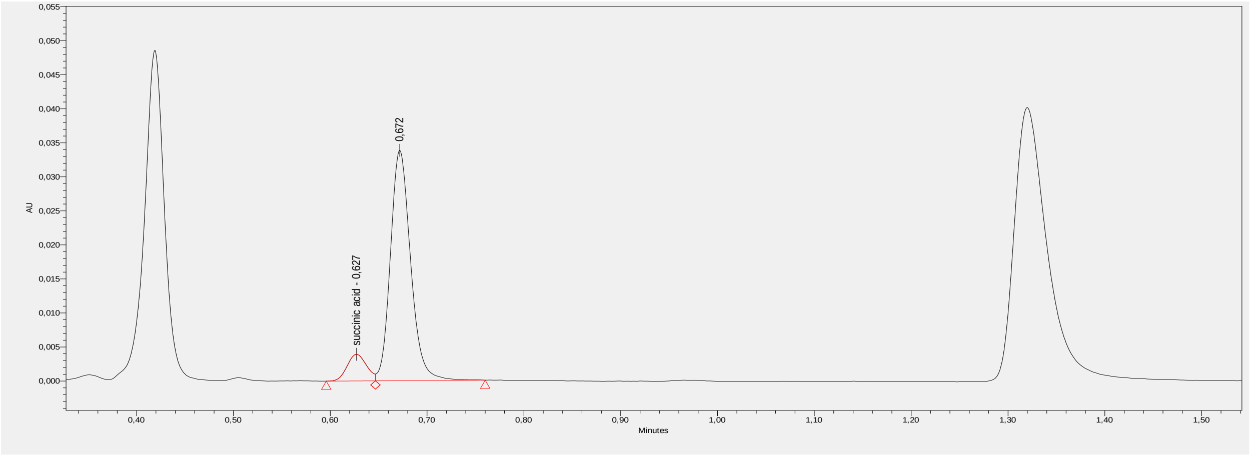

Figure S4.1: UHPLC traces of mPEG$_{5000}$-b-P(HPMAmLac$_n$-co-HPMAmLac$_n$-ETSA) polymer P100-E15 after NaOH hydrolysis for the quantification of succinic acid to determine ETSA content.

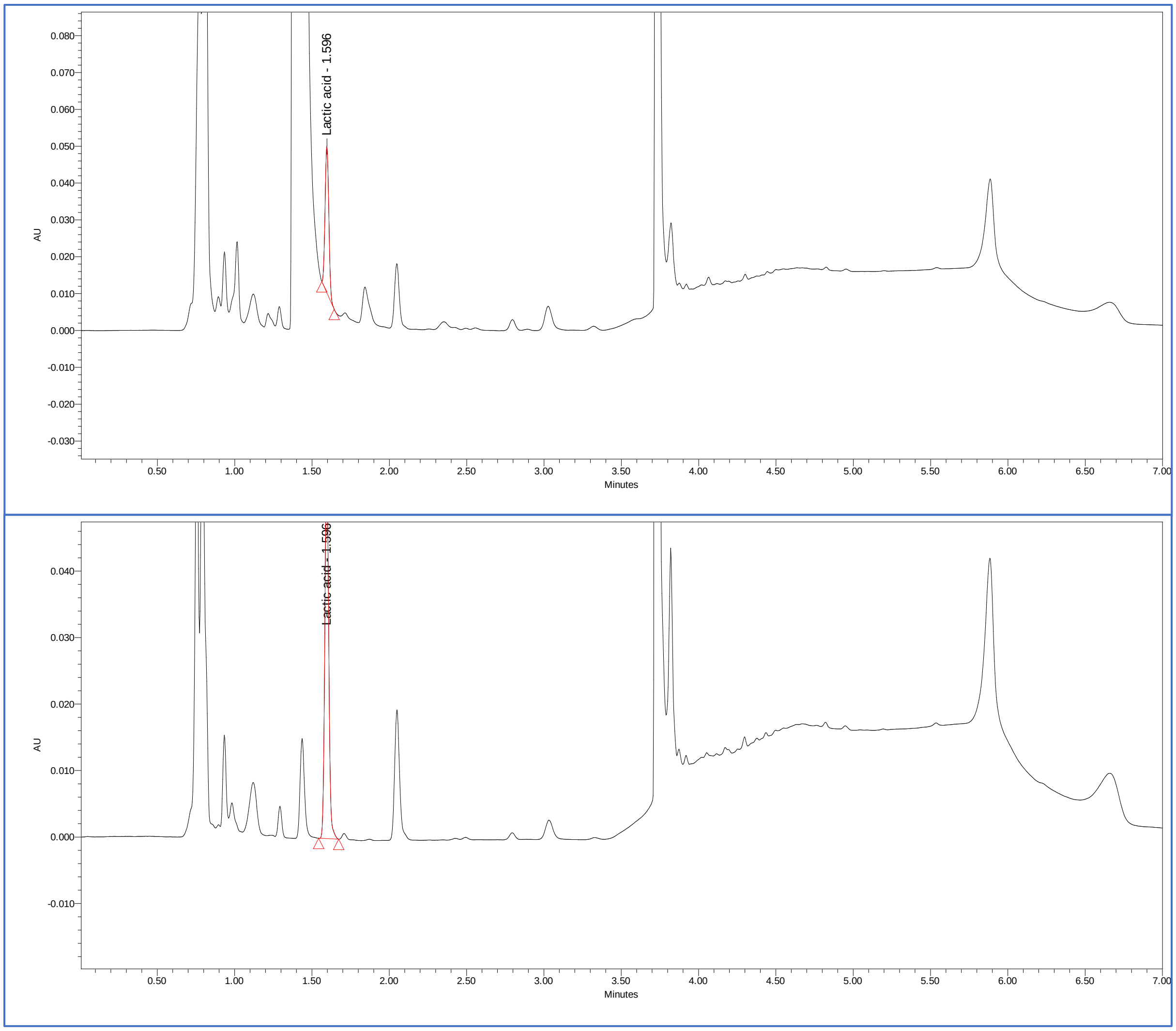

Figure S4.2: UHPLC traces before (top) and after (bottom) purification of CCPMs formed from mPEG$_{5000}$-b-P(HPMAmLac$_n$-co-HPMAmLac$_n$-ETSA) polymer P100-E15. The CCPMs were hydrolyzed in NaOH for the quantification of lactic acid to determine the total polymer content. The purified CCPM sample was found to have a polymer concentration of 7.5 mg/mL which is very close to the expected value of 7.0 mg/mL, based on dilution. (indicating no significant losses during purification). The crude sample resulted in an underestimate of 15.4 mg/mL out of the expected 18.5 mg/mL which is attributed to the interference of a neighboring peak (DMSO) with the lactic acid peak in the chromatogram.

5. Size Exclusion Chromatograms

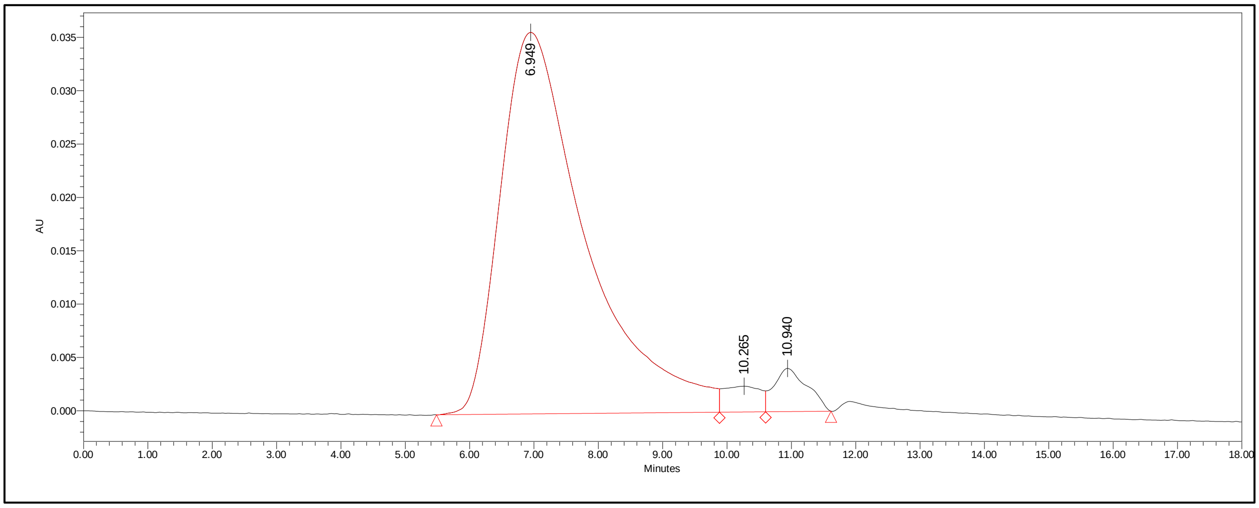

Figure S5.1: Size exclusion chromatogram of empty CCPMs. Detection at 235 nm.

6. Illustrative Figures

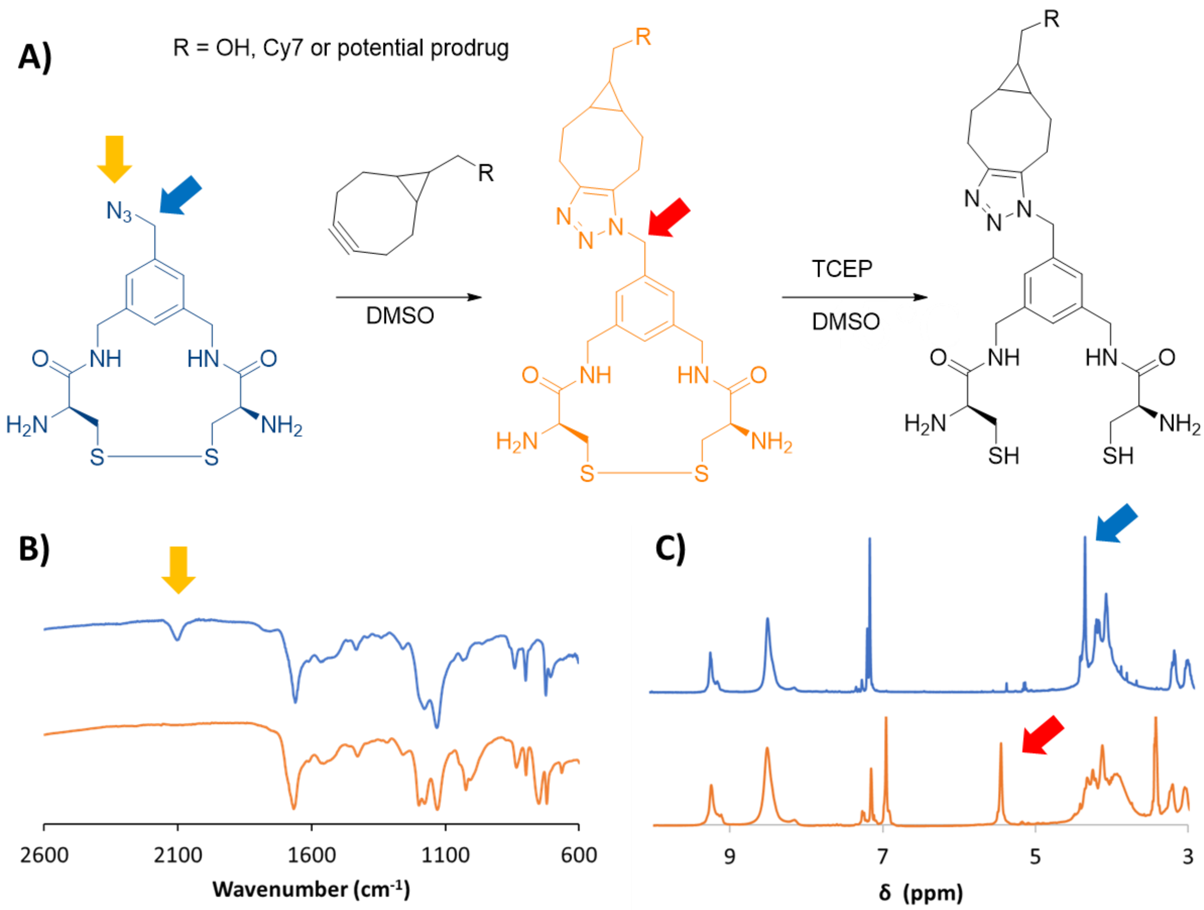

Figure S6.1: A) Schematic representation of the reaction between the crosslinker azide and a BCN functionality. Azide and a BCN functionality followed by TCEP induced disulfide reduction. B) IR spectra of the crosslinker before (blue) and after (orange) the click reaction with BCN-OH. C) NMR spectra of the crosslinker before (blue) and after (orange) the click reaction with BCN-OH. The results shown indicate that the crosslinker-BCN reaction results in the anticipated triazole ring formation.

7. Flow cytometry

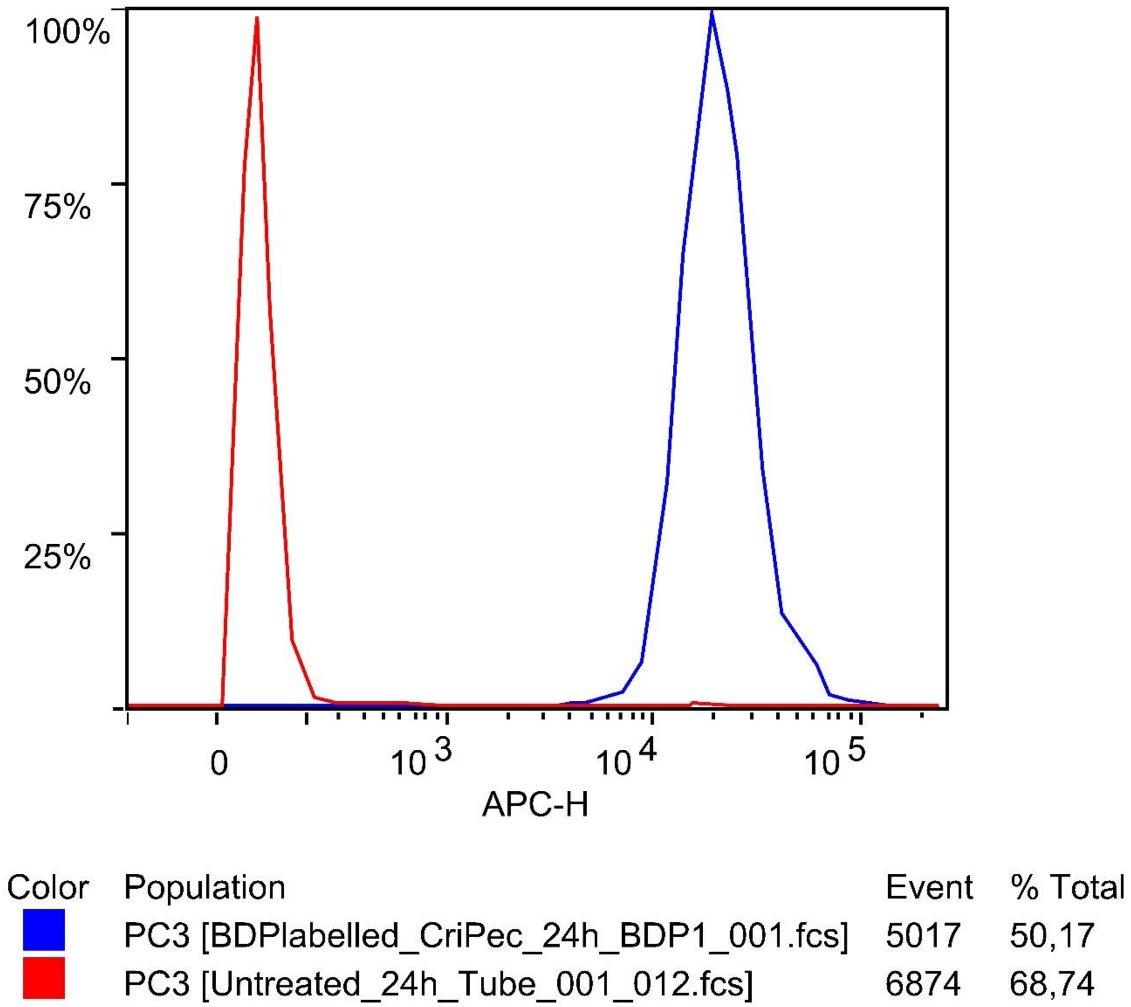

Figure S7.1: Cell associated fluorescence of PC3 cells incubated for 24 hours with BODIPY loaded CCPMs.
